# CircSLC3A2 functions as an oncogenic factor in hepatocellular carcinoma by sponging miR-490-3p and regulating PPM1F expression

**DOI:** 10.1186/s12943-018-0909-7

**Published:** 2018-11-23

**Authors:** Hongjian Wang, Wei Chen, Ming Jin, Lidan Hou, Xiaoyu Chen, Rui Zhang, Jing Zhang, Jinshui Zhu

**Affiliations:** 1 The Fifth Department of Digestion, The First Affiliated Hospital of Zhengzhou University, Zhengzhou, 450052 People’s Republic of China; 2 Department of Gastroenterology, Shanghai Jiao Tong University Affiliated Sixth People’s Hospital, No. 600 Yishan Road, Shanghai, 200233 China; 3 Department of Clinical Medicine, Ningbo University School of Medicine, Ningbo, 315211 Zhejiang Province China; 4 Department of Gastroenterology, Shanghai Ninth People’s Hospital, Shanghai Jiao Tong University School of Medicine, Shanghai, China

**Keywords:** PPM1F, CircSLC3A2, MiR-490-3p, Proliferation, Invasion, Hepatocellular carcinoma

## Abstract

**Background:**

Non-coding RNAs (ncRNAs) have been reported to participate in tumor progression by regulating gene expression. Previous studies showed that protein phosphatase Mg2^+^/Mn2^+^ dependent 1F (PPM1F) acts a dual role in cancer growth and metastasis. But, the underlying mechanisms by which ncRNAs regulate PPM1F expression in hepatocellular carcinoma (HCC) are poorly understood.

**Methods:**

The association between PPM1F or miR-490-3p expression and clinicopathological features and prognosis in patients with HCC was analyzed by TCGA RNA-sequencing data. CircSLC3A2 was identified to bind with miR-490-3p by bioinformatic analysis, and the binding sites between miR-490-3p and PPM1F or circSLC3A2 were confirmed by dual luciferase report and RNA immunoprecipitation (RIP) assays. The localization and clinical significance of miR-490-3p and circSLC3A2 in patients with HCC were investigated by fluorescence in situ hybridization (FISH). MTT, Agar, and Transwell assays were conducted to evaluate the effects of miR-490-3p or circSLC3A2 on cell proliferation and invasive potential.

**Results:**

The expression of PPM1F or miR-490-3p was associated with poor survival and tumor recurrence, and acted as an independent prognostic factor in patients with HCC. Re-expression of miR-490-3p inhibited HCC cell proliferation and invasion by targeting PPM1F, but its inhibitor reversed these effects. Moreover, circSLC3A2, predominantly localized in the cytoplasm, exhibited an oncogenic role by sponging miR-490-3p and regulating PPM1F expression, and harbored a positive correlation with poor survival in patients with HCC.

**Conclusion:**

CircSLC3A2 acts as an oncogenic factor in HCC by sponging miR-490-3p and regulating PPM1F expression.

**Electronic supplementary material:**

The online version of this article (10.1186/s12943-018-0909-7) contains supplementary material, which is available to authorized users.

## Introduction

Hepatocellular carcinoma (HCC) is the second leading cause of cancer-related death and its incidence is increasing each year worldwide [1]. Despite the feasible and effective therapies for HCC, such as surgical resection, ablation, chemotherapy and liver transplantation, the prognosis of patients with HCC remains very poor duo to its aggressiveness and diagnosis at a late stage [2]. HCC is a long-term progressive disease with multiple genetic alterations, leading to the activation of oncogenes or inactivation of tumor suppressor genes [3]. Therefore, identification of novel promising biomarkers may provide insights into the early diagnosis of HCC.

Protein phosphatase, Mg2^+^/Mn2^+^ dependent 1F (PPM1F), a member of the PP2C family of Ser/Thr protein phosphatases, has been reported to regulate cancer cell apoptosis, proliferation and metastasis. PPM1F, expressed in a variety of tumor cell lines, facilitates cell motility and invasiveness [4] and breast carcinogenesis by inactivating p53 signaling [5], promotes tumor metastasis via MAPK signaling and exosome cytokine secretion [6], and represses cell apoptosis through the TAK1-IKK-NF-κB pathway [7]. However, our previous study showed that PPM1F is downregulated in gastric cancer (GC), and low expression of PPM1F is associated with poor survival in patients with GC [8].

MicroRNAs (miRNAs) are a subgroup of endogenous non-coding RNAs (ncRNAs) with 20~ 25 nucleotides in length and function by negatively regulating their target genes [9]. Accumulating evidence indicates that miRNAs can act as oncogenic or suppressive factors involved in the progression of HCC [10–12], and some even exhibit antitumor effects by regulating PPM1F expression. For example, miR-200c and miR-149 inhibit the invasion and metastasis in breast cancer and HCC by targeting PPM1F [13, 14], but miR-590 has the tumor promoting effects in GC by targeting PPM1F [8].

Circular RNAs (circRNAs) as another new class of ncRNAs possess a closed loop and higher conservatism than linear RNA duo to their resistance to RNase R [15]. Increasing data show that circRNAs, predominantly localized in the cytoplasm, can act as the miRNA sponges to participate in the pathogenesis of HCC [16]. CircRNA cSMARCA5 and circMTO1, associated with tumor aggressive characteristics, suppress the growth and metastasis of HCC by sponging miR-17-3p, miR-181b-5p and miR-9 [17, 18], whereas circ_0067934 favors the growth and metastasis of HCC by regulating miR-1324/FZD5/Wnt/β-catenin signaling [19]. These studies suggest that circRNAs may represent novel biomarkers for the diagnosis and treatment of HCC.

In this study, we found that the expression of PPM1F or miR-490-3p was associated with poor survival and tumor recurrence, and acted as an independent prognostic factor in patients with HCC. Then, miR-490-3p was identified to have a negative correlation with PPM1F expression and inhibited cell proliferation and invasion by targeting PPM1F. CircSLC3A2 was further found to function as an oncogenic factor in HCC cells by sponge miR-490-3p and regulating PPM1F expression, and was associated with the poor survival in patients with HCC.

## Materials & methods

### Clinical data

The clinicopathological data of 50 paired HCC and adjacent non-tumor tissues as well as 372 unpaired HCC tissues, the relative expression levels of PPM1F and miRNAs (has-miR-186-5p, has-miR-200c, has-miR-200b, has-miR-429, has-miR-425-5p and has-miR-490-3p) were downloaded from The Cancer Genome Atlas (TCGA)-Liver Cancer RNA sequencing database (https://genome-cancer.ucsc.edu). The tissue microarray (TMA) of 90 paired HCC patients (Cat No. K16–027) was purchased from the shanghai Outdo Biotech Company (Shanghai, PR, China). The protocols used in our study were approved by the Ethics Committee of Shanghai Sixth People’s Hospital. HCC specimens were classified according to the WHO criteria and TNM staging system, and the pathological diagnosis for these samples was accomplished by two independent pathologists.

### Identification of miRNAs that target PPM1F 3′UTR

The miRNAs that target PPM1F 3′UTR were identified using both the TargetScan_7.1 (http://www.targetscan.org/vert_71/) and microRNA.org (http://starbase.sysu.edu.cn/targetSite.php), and the intersecting miRNAs from these two prediction tools were used for further verification.

### Cell culture

Normal liver tissue and HCC cell lines (HepG2, Huh6, Huh7, SK-hep-1, SMMC-7721 and LO2) were from Cell bank of the Chinese Academy of Sciences and cultured in Dulbecco’s Modified Eagle medium (DMEM) medium supplemented with 10% heat-inactivated fetal bovine serum (FBS). Cells in this medium were placed in a humidified atmosphere containing 5% CO_2_ at 37 °C.

### RNA fluorescence in situ hybridization (FISH)

Oligonucleotide modified probe sequences for miR-490-3p (5′-CY3-CAGCATGGAG TCCTCCAGGTTG-CY3–3) and circSLC3A2 (5′-FAM-GTAGTTGGGAGTAAG GT CCAGAATGACACGGATGCCTGTCCAGGAA-FAM-3′.) were used for FISH. The detailed experimental process was performed as previously described [20]. The analysis software Image-pro plus 6.0 (Media Cybernetics, Inc., Rockville, MD, USA) was used to analyse the Immunofluorescence Accumulation Optical Density (IOD) of miR-490-3p and circSLC3A2 in HCC tissues.

### Quantitative real-time PCR (qRT-PCR)

Total RNA was extracted using TRIzol and reverse transcription was conducted using M-MLV and cDNA amplification using the SYBR Green Master Mix kit (Takara, Otsu, Japan). In addition, total RNA was separated using a High Pure miRNA isolation kit (Roche) and RT-PCR using a TaqMan MicroRNA Reverse Transcription kit (Life Technologies). The nuclear and cytoplasmic fractions were isolated using NE-PER Nuclear and Cytoplasmic Extraction Reagents (Thermo Scientific). The primer sequences were indicated in Additional file 1: Table S1.

### Western blotting analysis

HepG2 and LO2 cell lines were harvested and extracted using lysis buffer. Cell extracts were boiled in loading buffer and equal amount of cell extracts were separated on 15% SDS-PAGE gels. Separated protein bands were transferred into polyvinylidene fluoride (PVDF) membranes. The primary antibodies against PPM1F (ab156222, Rabbit polyclonal antibody, Abcam, Cambridge, MA, USA) and β-actin (ab16039, Rabbit polyclonal antibody, Abcam, Cambridge, MA, USA) were diluted at a ratio of 1:1000 according to the instructions and incubated overnight at 4 °C. Horseradish peroxidase-linked secondary antibodies were added at a dilution ratio of 1:10000, and incubated at room temperature for 1 h. The membranes were washed with PBS for three times and the immunoreactive bands were visualized using ECL-PLUS/Kit (GE Healthcare, Piscataway, NJ, USA) according to the kit’s instruction.

### Luciferase reporter assay

HepG2 and LO2 cell lines were seeded into 96-well plates and were co-transfected with a mixture of 60 ng of luciferase, 6 ng of pRL-CMV Renilla luciferase reporter, and miR-490-3p mimic or inhibitor. After 48 h of incubation, the firefly and Renilla luciferase activities were measured with a dual-luciferase reporter assay (Promega, Madison, WI, USA).

### Plasmid, shRNA, miRNA mimic and inhibitor

Plasmid mediated PPM1F or circSLC3A2 vector, RNAi targeting PPM1F or circSLC3A2 vector, miR-490-3p mimic and inhibitor were purchased from GenePharma (Shanghai, PR, China) and the negative control (NC), pcDNA3.1 or miR-NC was used as the control vector. Designed RNAi sequences were listed as follows: si-PPM1F (NM_014634_siRNA_405): GCTGCTACAGACAGACCTT and NM_ 014634_siRNA_control_405: GCTACATACAGCAGCGCTT; sh-circSLC3A2: GAC AGGCATCCGTGTCATTCT and sh-NC: GCTCACTTAGTTATCGGAC. HepG2 and LO2 cell lines were planted in 6-well plates 24 h prior to si-PPM1F, sh-circSLC3A2, miR-490-3p mimic or inhibitor transfection with 50–60% confluence, and then were transfected with Lipofectamine 2000 (Invitrogen, Carlsbad, CA, USA) according to the manufacture instructions.

### MTT and Transwell assays

MTT assay for cell viability and Transwell assay for cell invasion were conducted as previously described [20].

### Agar assay

Cells were trypsinized, and 5000 cells were resuspended in 2 × medium plus 0.7% agar (Sigma). The agar–cell mixture was plated on top of a bottom layer consisting of 1.2% agar in complete medium. After 10 days, colony size was measured using an ocular micrometer and colonies larger than 0.1 mm in diameter were counted. The experiment was performed three times for each cell line.

### RNase R treatment

Total RNA (2 μg) was incubated for 30 min at 37 °Cwith 3 U/μg of RNase R (Epicentre Technologies, Madison, WI, USA). After treatment with RNase R, the mRNA levels of SLC3A2 and circSLC3A2 were examined by qRT-PCR analysis.

### RNA immunoprecipitation (RIP)

RIP assay was performed using a Magna RIP RNA-binding protein Immunoprecipitation Kit (Millipore) according to the manufacturer’s instructions. Antibodies for RIP assays against Ago2 and IgG were purchased from Abcam (ab5072, Rabbit polyclonal antibody, Cambridge, MA, USA).

### Statistical analysis

Statistical analyses were implemented using SPSS 20.0 (IBM, SPSS, Chicago, IL, USA) and GraphPad Prism. Student’s t-test and Chi-square test were used to analyze the statistical significance for comparisons of two groups. The Pearson’s correlation coefficient analysis was used to observe the correlations between miRNAs and PPM1F or circSLC3A2 expression in HCC tissues. Overall survival and disease-free survival (DFS or recurrence) curves were analyzed with Kaplan-Meier method and log-rank test. Univariate analysis and multivariate models were conducted using a Cox proportional hazards regression model. Cutoff value and Receiver operating characteristic (ROC) curve were obtained using cutoff finder online software (http://molpath.charite.de/cutoff/load.jsp). *P* < 0.05 was considered statistically significant.

## Results

### Upregulation of PPM1F expression was associated with poor survival in patients with HCC

**Fig. 1:**
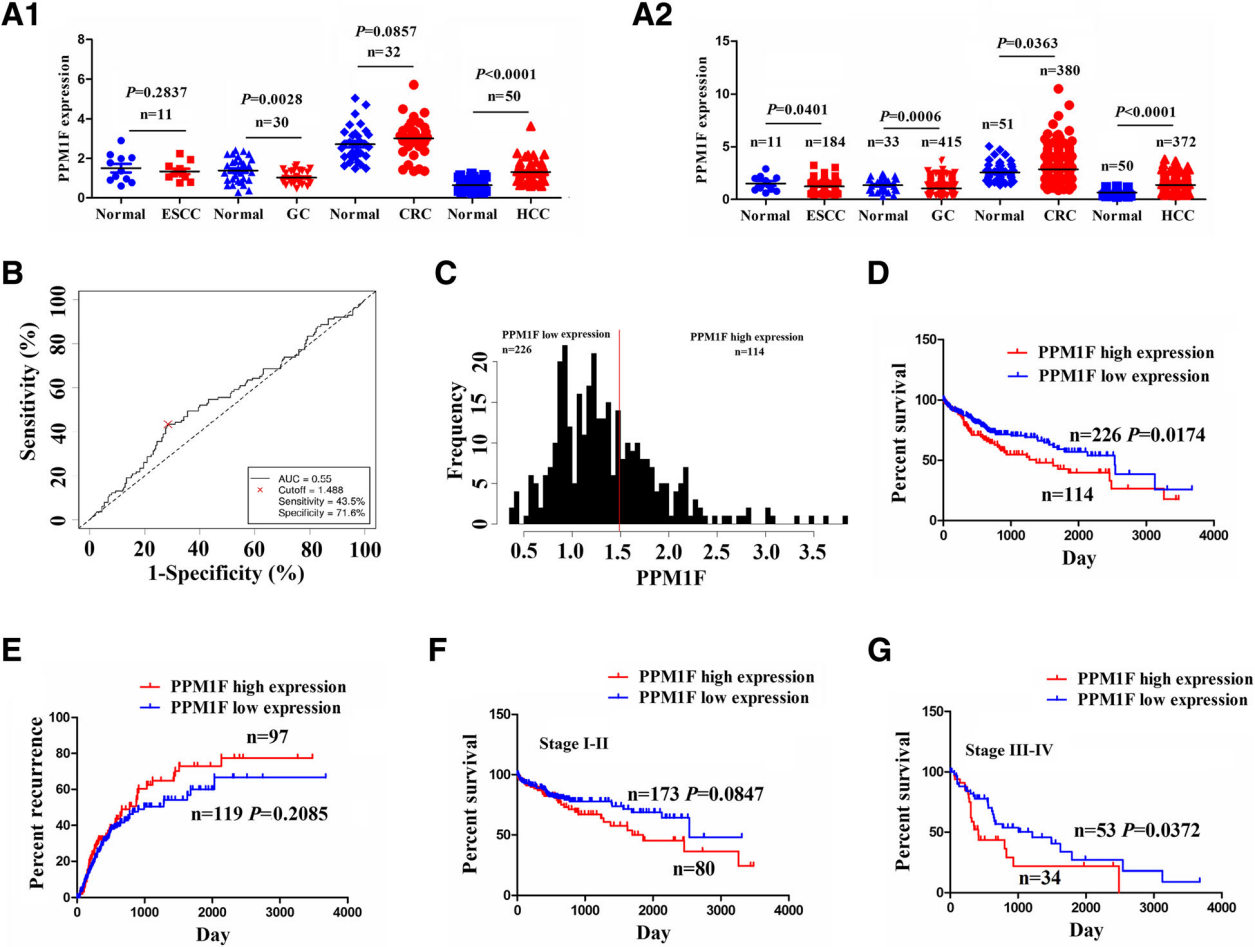
The association between PPM1F expression and the prognosis in patients with HCC. (**a1–2**) TCGA cohort analysis of the expression level of PPM1F in paired and unpaired HCC tissues. **b** ROC curve analysis of the cutoff value, sensitivity, specificity and AUC of PPM1F in HCC tissues. **c** The patients with HCC were divided into high or low PPM1F expression according to its cutoff value. **d**, **e** Kaplan Meier analysis of the association of high or low PPM1F expression with the overall survival and tumor recurrence in patients with HCC. **f**, **g** Kaplan Meier analysis of the association of high or low PPM1F expression with the survival in patients with HCC in early and late stages

We examined the expression levels of PPM1F in multiple gastrointestinal tumors using the TCGA database and found that PPM1F had the most obvious upregulation in paired (*n* = 50, *P* < 0.0001; Fig. 1a1) and unpaired HCC tissues (*n* = 372, *P* < 0.0001; Fig. 1a2) as compared with the other tumor tissues. To assess the clinical significance of PPM1F in HCC, we analyzed the association between PPM1F expression and clinicopathological characteristics and prognosis in patients with HCC. As shown in Fig. 1b, the cutoff value of PPM1F (1.488) was acquired according to its expression levels, survival time and survival status in patients with HCC, and the patients were divided into high PPM1F expression and low PPM1F expression groups (Fig. 1c).

As shown in Additional file 1: Table S2, high expression of PPM1F had no association with the clinicopathological factors (each *P* > 0.05). Kaplan Meier analysis showed that HCC patients with high PPM1F expression harbored poorer survival (*P* = 0.0174, Fig. 1d), but had no difference in tumor recurrence (*P* = 0.2085, Fig. 1e) as compared with those with low PPM1F expression. On the basis of TNM stage, the patients in late stage (stage III + IV, *P* = 0.0372) rather than early stage (stage I + II, *P* = 0.0847) with high PPM1F expression possessed poorer survival as compared with those with low PPM1F expression (Fig. 1f, g). Furthermore, univariate and multivariate Cox regression analysis revealed that PPM1F expression as well as distant metastasis was an independent prognostic factor of poor survival in patients with HCC (Additional file 1: Table S3).

### MiR-490-3p displayed a negative correlation with PPM1F expression in HCC tissues

To elucidate the reason of PPM1F upregulation in HCC tissues, we investigated the alterations of PPM1F in genetic or epigenetic levels, and found little evidence about the dysregulation of PPM1F at the genetic (Additional file 2: Figure S1A and B) and methylation levels (Additional file 2: Figure S1C), indicating that genetic alterations and methylation modification could not account for the upregulation of PPM1F in HCC.

**Fig. 2:**
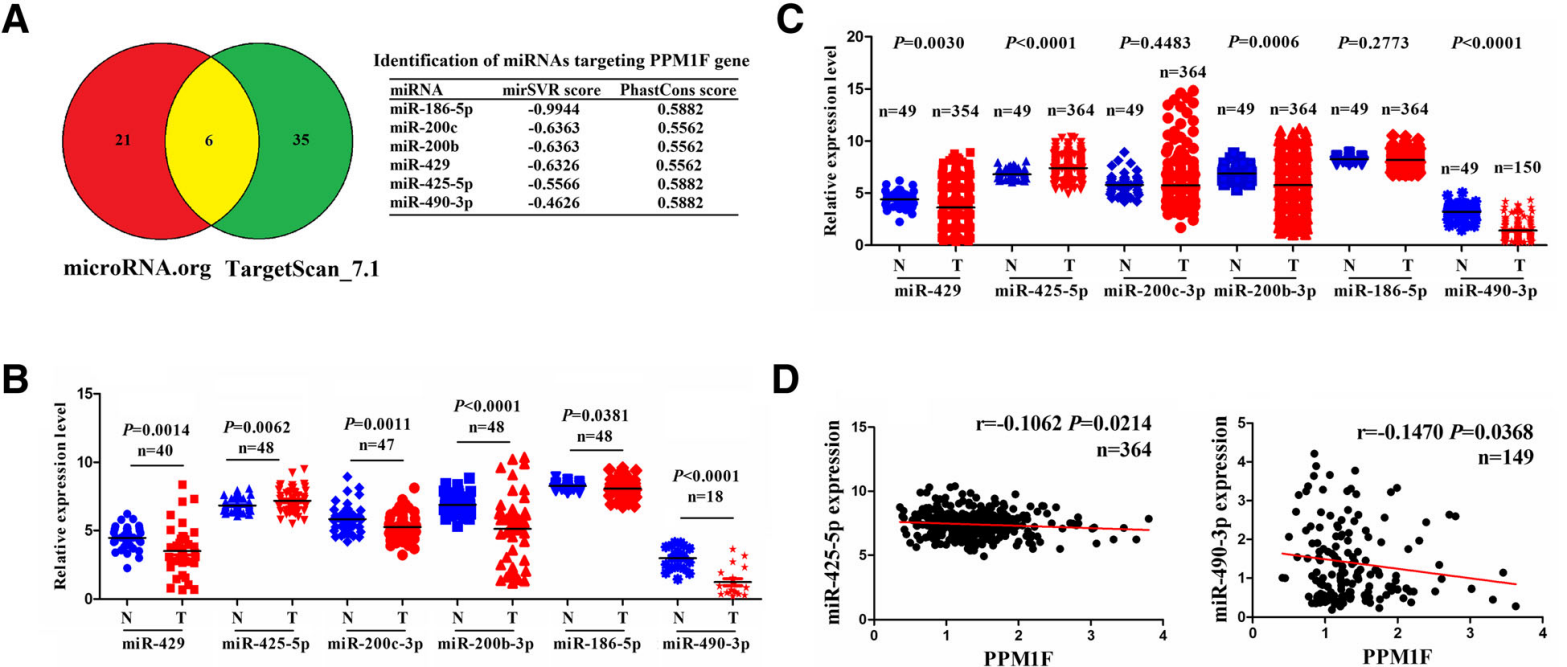
Identification of the correlation of PPM1F expression with miRNAs in patients with HCC. **a** Identification of the miRNAs that target PPM1F 3′UTR by microRNA.org and Targetscan_7.1 prediction tools. **b**, **c** TCGA cohort analysis of the expression levels  of miRNAs in paired and unpaired HCC tissues. **d** Pearson’s correlation coefficient analysis of the correlation of PPM1F expression with miR-425-5p and miR-490-3p in HCC tissues

Whether PPM1F upregulation was caused by miRNAs through the post-transcriptional regulation is needed to be clarified. TargetScan_7.1 and microRNA.org prediction tools were used to identify six miRNAs that may have the potential to bind to PPM1F 3’ UTR (Fig. 2a and Additional file 1: Table S4, S5). The expression levels of these miRNAs were then examined in paired (Fig. 2b) and unpaired HCC tissues (Fig. 2c), indicating that miR-490-3p had the most significant downregulation in HCC tissues (*P* < 0.0001). Pearson’s coefficient analysis showed that miR-490-3p rather than other five miRNAs had the most obviously negative correlation with PPM1F expression (*r* = − 0.1470, *P* = 0.0368; Fig. 2d and Additional file 3: Figure S2A1–4) and was selected for further observation.

### Low expression of miR-490-3p was associated with poor prognosis in patients with HCC

**Fig. 3:**
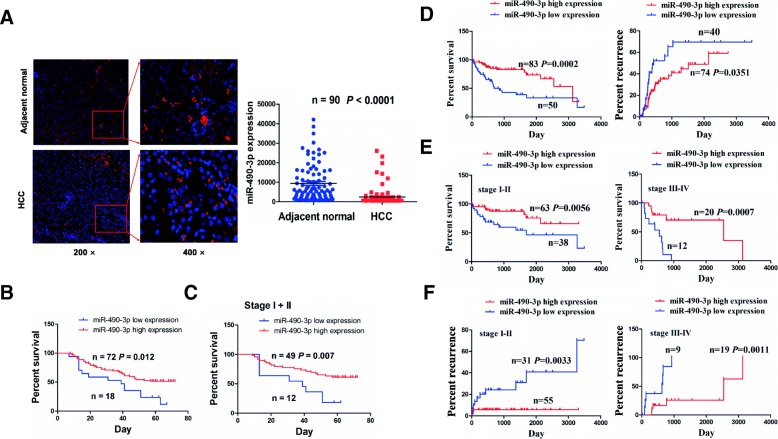
The association between miR-490-3p expression and the prognosis in patients with HCC. **a** FISH analysis of the expression level of miR-490-3p in HCC and adjacent normal tissues. **b**, **c** Kaplan-Meier analysis of the association of high or low miR-490-3p expression with the overall survival in patients with HCC or those in early stage. **d** Kaplan-Meier analysis of the association of high or low miR-490-3p expression with the overall survival and tumor recurrence in patients with HCC in TCGA cohort. **e**, **f** Kaplan-Meier analysis of the association of high or low miR-490-3p expression with the overall survival and tumor recurrence in HCC patients in early and late stages

We further detected the expression levels of miR-490-3p in HCC tissues by FISH and found that miR-490-3p was principally localized in the cytoplasm and dramatically downregulated in HCC tissues as compared with the adjacent non-tumor tissues (*P* < 0.0001, Fig. 3a). As shown in Additional file 3: Figure S2B, the cutoff value of miR-490-3p was obtained in our cohort, and we found that, HCC patients with low miR-490-3p expression displayed poorer survival as compared with those with high miR-490-3p expression (*P* = 0.012, Fig. 3b). The patients in early stage (*P* = 0.007, Fig. 3c) rather than in late stage (*P* = 0.599, Additional file 3: Figure S2C) with low miR-490-3p expression showed the similar survival prognosis. In TCGA cohort, the cutoff value, sensitivity, specificity and AUC of miR-490-3p were respectively 0.787, 73.3, 59.1% and 0.63 (Additional file 3: Figure S2D), indicating that miR-490-3p was a promising marker in HCC patients.

As shown in Additional file 1: Table S6, low expression of miR-490-3p had no association with the clinicopathological factors (each *P* > 0.05) in patients with HCC. Kaplan Meier analysis indicated that HCC patients with low miR-490-3p expression possessed poorer survival (*P* = 0.0002) and higher tumor recurrence (*P* = 0.0351) as compared with those with high miR-490-3p expression (Fig. 3d). The patients in early or late stage with low miR-490-3p expression also had poorer survival (*P* = 0.0056, *P* = 0.0007; Fig. 3e) and higher tumor recurrence (*P* = 0.0033, *P* = 0.0011; Fig. 3f) as compared with those with high miR-490-3p expression. Moreover, univariate and multivariate Cox regression analysis showed that miR-490-3p expression as well as pathological stage and TNM stage was an independent prognostic factor of poor survival and tumor recurrence in patients with HCC (Additional file 1: Table S7, S8).

### PPM1F was identified as a direct target of miR-490-3p in HCC cells

**Fig. 4:**
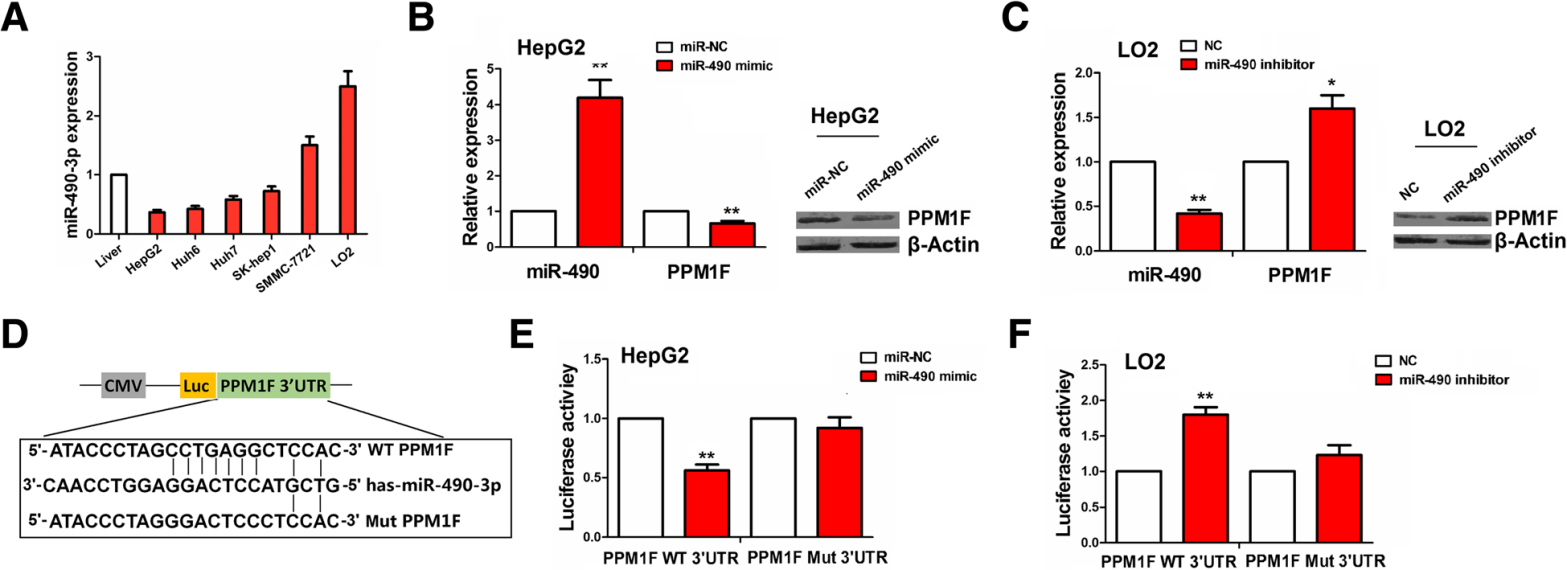
Identification of PPM1F as a direct target of miR-490-3p in HCC cells. **a** qRT-PCR analysis of the expression levels of miR-490-3p in different HCC cell lines. **b**, **c** qRT-PCR and Western blot analysis of the expression levels of miR-490-3p and PPM1F after transfection with miR-490-3p mimic in HepG2 cell line or its inhibitor in LO2 cell line. **d** The binding sites of miR-490-3p with WT or Mut PPM1F 3′UTR. **e**, **f** Luciferase activity of WT or Mut PPM1F 3′UTR after transfection with miR-490-3p mimic or inhibitor in HepG2 or LO2 cell line. Data are the means ± SEM of three experiments. * *P* < 0.05; ** *P* < 0.01

We examined the expression levels of miR-490-3p in HCC cell lines by qRT-PCR analysis, indicating that miR-490-3p had lower expression in HepG2 cell line (*P* < 0.01), but higher expression in LO2 cell line (*P* < 0.05) as compared with the normal liver tissue (Fig. 4a). Then, qRT-PCR and western blot analysis showed that miR-490-3p expression was substantially increased, but PPM1F expression was decreased by transfection with miR-490-3p mimic in HepG2 cells (**P < 0.01, Fig. 4b), whereas the opposite results were indicated by transfection with miR-490-3p inhibitor in LO2 cells (*P < 0.05, **P < 0.01; Fig. 4c). Luciferase reporter vectors containing wild type (WT) or mutant (Mut) PPM1F 3′UTR (Fig. 4d) in combination with miR-490-3p mimic or inhibitor were transfected into HepG2 or LO2 cell line. The results showed that the luciferase activity of WT PPM1F 3′UTR was decreased by miR-490-3p mimic in HepG2 cell line (**P < 0.01, Fig. 4e), but increased by miR-490-3p inhibitor in LO2 cell line as compared with the control group (**P < 0.01, Fig. 4f). However, the luciferase activity of Mut PPM1F 3′UTR was not affected by miR-490-3p mimic or inhibitor in HepG2 or LO2 cells as compared with the control group (*P* > 0.05, Fig. 4e, f).

### PPM1F reversed the inhibitory effects of miR-490-3p in HCC cells

**Fig. 5:**
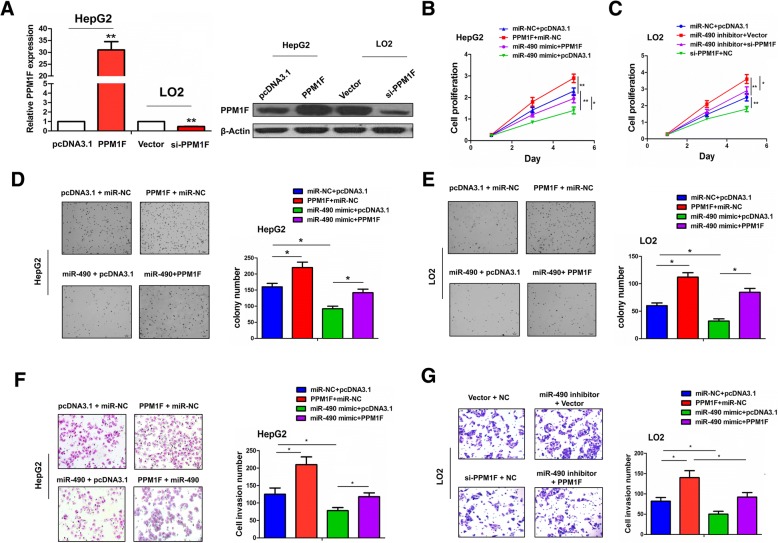
MiR-490-3p inhibited HCC cell proliferation and invasion by regulating PPM1F expression. **a** qRT-PCR and Western blot validation of the transfection efficiency of PPM1F or si-PPM1F plasmid in HepG2 or LO2 cell line. **b**, **c** MTT analysis of the cell viability after transfection with miR-490-3p mimic and PPM1F plasmid in HepG2 cell line or miR-490-3p inhibitor and si-PPM1F plasmid in LO2 cell line. **d**, **e** Agar assay analysis of the anchorage-independent growth after transfection with miR-490-3p mimic and PPM1F plasmid in HepG2 cell line or miR-490-3p inhibitor and si-PPM1F plasmid in LO2 cell line. **f**, **g** Transwell analysis of the cell invasive potential after transfection with miR-490-3p mimic and PPM1F plasmid in HepG2 cell line or miR-490-3p inhibitor and si-PPM1F plasmid in LO2 cell line. Data are the means ± SEM of three experiments. * *P* < 0.05; ** *P* < 0.01

To further define the effects of miR-490-3p on PPM1F expression in HCC cells, we conducted the functional experiments such as MTT, Agar and Transwell assays. The expression levels of PPM1F were validated after transfection with PPM1F or si-PPM1F vector in HepG2 or LO2 cell lines by qRT-PCR and Western blot analysis (**P < 0.01, Fig. 5a). Cell viability was increased by PPM1F overexpression or miR-490-3p inhibitor, but decreased by knockdown of PPM1F or miR-490-3p mimic in HepG2 or LO2 cell line (*P < 0.05, **P < 0.01; Fig. 5b, c). Moreover, the anti-proliferative effect induced by miR-490-3p mimic was inversed by ectopic expression of PPM1F in HepG2 cells (*P < 0.05, Fig. 5b), but the proliferation-promoting effect caused by miR-490-3p inhibitor was counteracted by knockdown of PPM1F in LO2 cells (**P < 0.01, Fig. 5c). The similar phenomenon for the anchorage-independent growth (*P < 0.05, Fig. 5d, e), and cell invasion was shown in Fig. 5f, g (*P < 0.05).

### Identification of a new circSLC3A2 in HCC cells

**Fig. 6:**
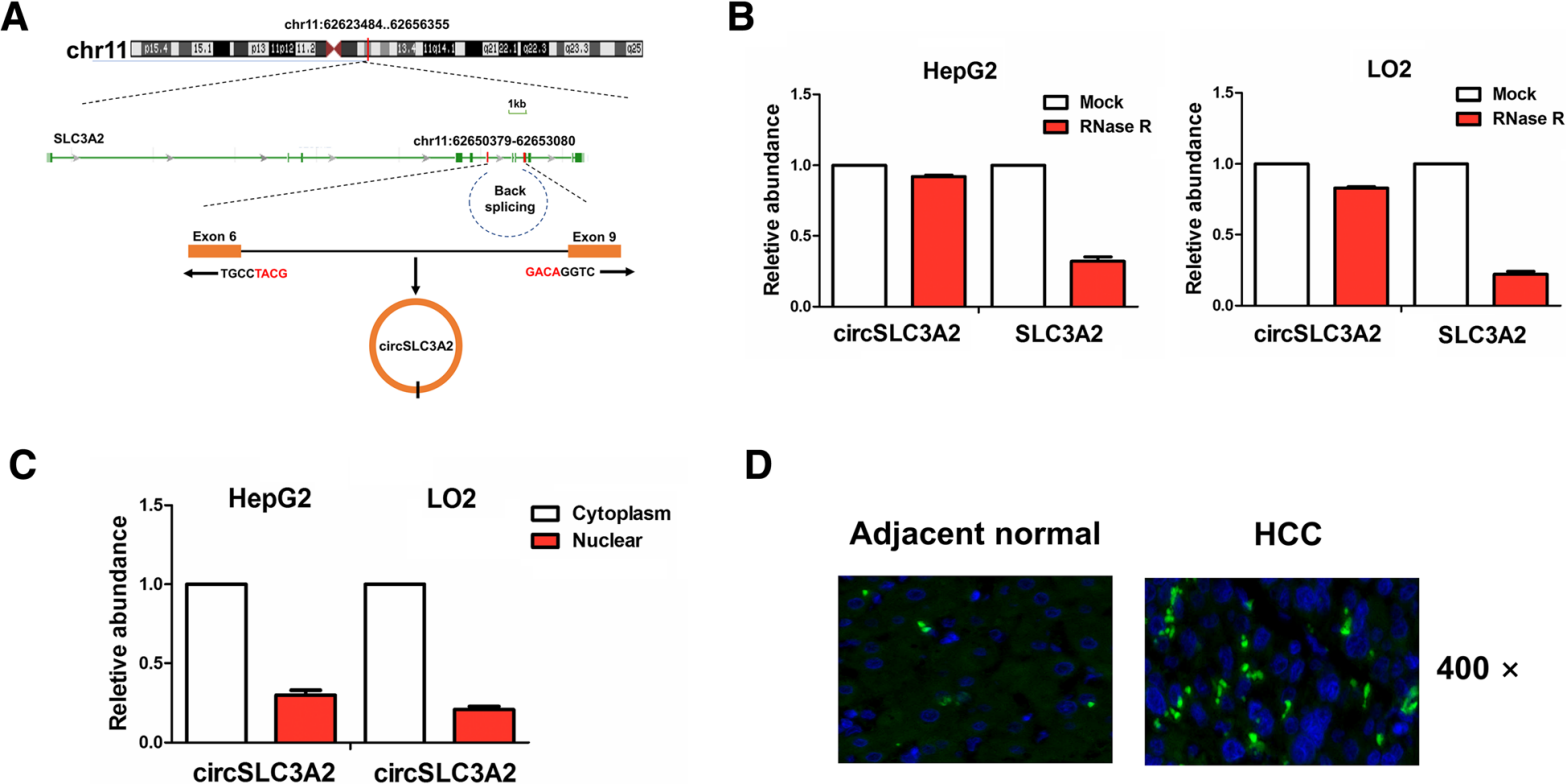
Identification of a new circSLC3A2 in HCC cells. **a** The genomic loci of the SLC3A2 gene and circSLC3A2. Arrows represent divergent primers that bind to the genomic region of circSLC3A2. **b** qRT-PCR analysis of the expression levels of circSLC3A2 and SLC3A2 after treatment with RNase R in HepG2 and LO2 cell lines. **c** qRT-PCR analysis of the expression of circSLC3A2 in the cytoplasm and nucleus of HepG2 and LO2 cell lines. **d** FISH analysis of the cellular localization of circSLC3A2 in HCC and adjacent normal tissue cells. The nuclei were stained with DAPI for blue color, and the positive expression of circSLC3A2 in the cytoplasm was stained for green color. Data are the means ± SEM of three experiments

To identify the circRNAs that can sponge miR-490-3p, we used the circRNAs expression profile and miRNAs prediction software screen out 9 circRNAs that had the potential to bind to the miR-490-3p (Additional file 1: Table S9 and Additional file 4: Figure S3), of which hsa_circ_0022587 might have the greatest potential to sponge miR-490-3p. It was noted that hsa_circ_0022587 is derived from exon 6, 9 regions within solute carrier family 3 member 2 (SLC3A2) locus, and is named as circSLC3A2 (Fig. 6a). According to the qRT-PCR analysis, circSLC3A2 exhibited a resistance to digestion induced by RNase R exonuclease as compared with the linear SLC3A2 in HepG2 and LO2 cell lines (*P* < 0.01, Fig. 6b). Cytoplasmic and nuclear RNA analysis showed that circSLC3A2 was preferentially localized in the cytoplasm in HepG2 and LO2 cell lines (Fig. 6c), and the consistent result was shown by FISH in HCC and adjacent normal tissue cells (Fig. 6d).

### CircSLC3A2 promoted cell proliferation and invasion in HCC cells

**Fig. 7:**
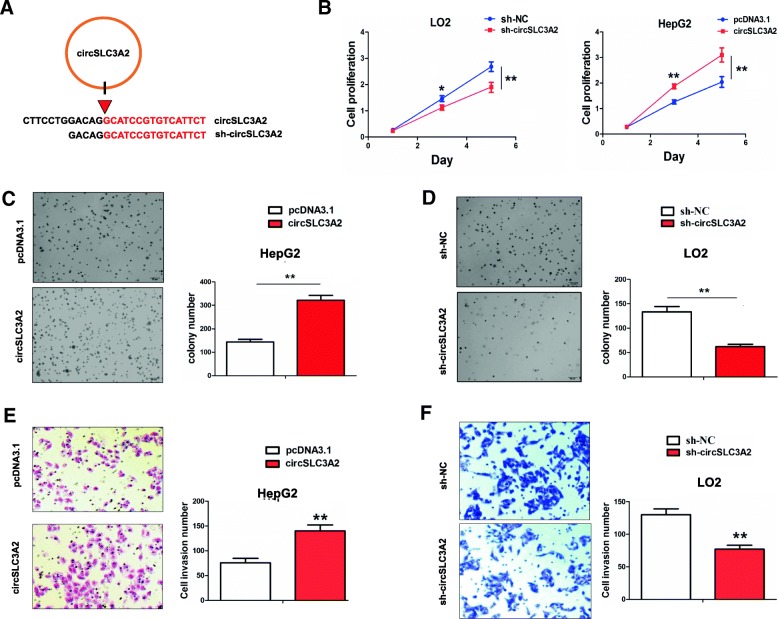
The effects of circSLC3A2 on HCC cell proliferation and invasion. **a** Schematic representation of target sequences of shRNA specific to the back-splicing junction of circSLC3A2. **b** MTT, **c**, **d** Agar assay and **e**, **f** Transwell analysis of the cell viability, anchorage-independent growth and the invasive potential after transfection with circSLC3A2 in HepG2 cells or sh-circSLC3A2 in LO2 cells. Data are the means ± SEM of three experiments. * *P* < 0.05; ** *P* < 0.01

We devised circSLC3A2 overexpression and shRNA sequences against the back-splicing site of circSLC3A2 (Fig. 7a). The transfection efficiency of circSLC3A2 or sh-circSLC3A2 in HepG2 or LO2 cell line was determined by qRT-PCR analysis (**P < 0.01, Additional file 5: Figure S4). MTT, Agar and Transwell assays indicated that cell viability (**P < 0.01, Fig. 7b), anchorage-independent growth (**P < 0.01, Fig. 7c, d) and invasive potential (**P < 0.01, Fig. 7e, f) were obviously enhanced by ectopic expression of circSLC3A2 in HepG2 cell line, but attenuated by knockdown of circSLC3A2 in LO2 cell line as compared with the control group.

### CircSLC3A2 acted as a sponge of miR-490-3p in HCC cells

**Fig. 8:**
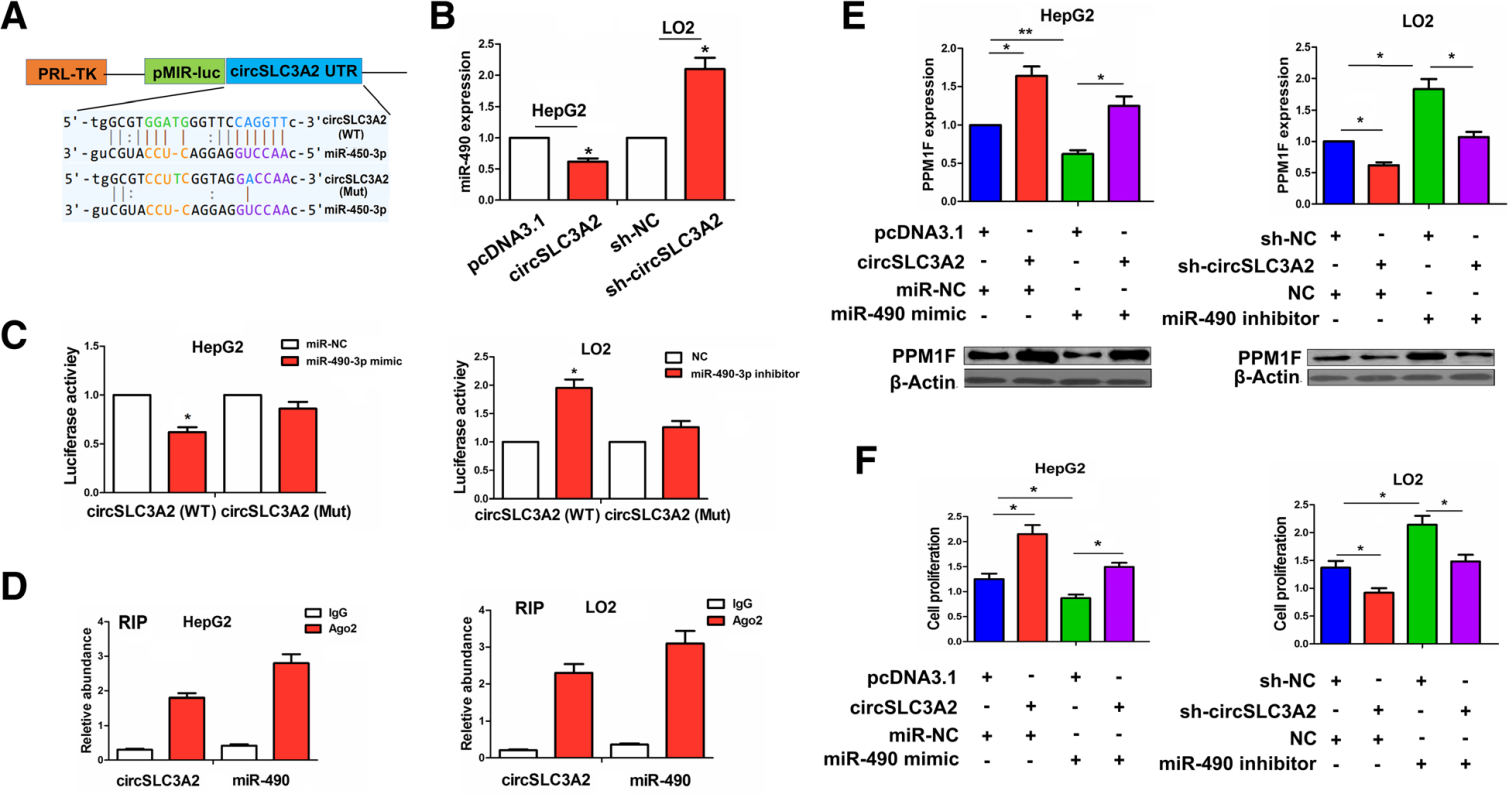
CircSLC3A2 acted as a sponge of miR-490-3p in HCC cells. **a** Schematic representation of potential binding sites of miR-490-3p with WT or Mut circSLC3A2. **b** qRT-PCR analysis of the expression level of miR-490-3p after transfection with circSLC3A2 in HepG2 cells or sh-circSLC3A2 in LO2 cells. **c** The luciferase activity of WT or Mut circSLC3A2 after co-transfection with pMIR-Luc-circSLC3A2 and miR-490-3p mimic or inhibitor in HepG2 or LO2 cell line. **d** Ago2 RIP assay for the amount of circSLC3A2 and miR-490-3p in HepG2 or LO2 cell line. **e** qRT-PCR and Western blot analysis of the expression levels of PPM1F after co-transfection with circSLC3A2 and miR-490-3p mimic in HepG2 cells or sh-circSLC3A2 and miR-490-3p inhibitor in LO2 cells. **f** MTT analysis of the cell viability after co-transfection with circSLC3A2 and miR-490-3p mimic in HepG2 cells or sh-circSLC3A2 and miR-490-3p inhibitor in LO2 cells. Data are the means ± SEM of three experiments. *P < 0.05; **P < 0.01

We established a circSLC3A2 fragment and incorporated it into downstream of the luciferase reporter gene (Fig. 8a). qRT-PCR analysis showed that the expression levels of miR-490-3p were decreased by overexpression of circSLC3A2 in HepG2 cells, but increased by knockdown of circSLC3A2 in LO2 cells (*P < 0.05, Fig. 8b). The luciferase reporter vector for pMIR-Luc-circSLC3A2 was co-transfected with miR-490-3p mimic or inhibitor into HepG2 or LO2 cell line. The luciferase activity of WT circSLC3A2 was decreased by miR-490-3p mimic in HepG2 cells, but increased by miR-490-3p inhibitor in LO2 cells (*P < 0.05, Fig. 8c). However, the luciferase activity of Mut circSLC3A2 was not affected by miR-490-3p mimic or inhibitor in HepG2 or LO2 cell line (*P* > 0.05). Furthermore, online circular RNA interactome (https://circinteractome.nia.nih.gov/index.html) was used to reveal a high degree of Ago2 occupancy in the region of circSLC3A2 (Additional file 1: Table S10). To validate this result, we conducted RIP assay for Ago2 in HepG2 and LO2 cell lines and examined the expression levels of endogenous circSLC3A2 and miR-490-3p pulled-down from Ago2-expressed cells by qRT-PCR analysis, indicating that circSLC3A2 and miR-490-3p expression was highly enriched in Ago2 pellet as compared with those in the input control (*P* < 0.01, Fig. 8d). We also investigated the expression of PPM1F, a target of miR-490-3p after co-transfection with circSLC3A2 and miR-490-3p mimic into HepG2 cell line or sh-circSLC3A2 and miR-490-3p inhibitor into LO2 cell line by qRT-PCR and Western blot analysis (*P < 0.05, **P < 0.01; Fig. 8e), indicating that circSLC3A2 overexpression increased the expression of PPM1F, and miR-490-3p mimic reversed this effect; Inversely, knockdown of circSLC3A2 decreased the expression of PPM1F and miR-490-3p inhibitor counteracted this effect. In addition, cell viability, increased by circSLC3A2 overexpression was reversed by miR-490-3p mimic in HepG2 cell line, while cell viability, decreased by knockdown of circSLC3A2 was counteracted by miR-490-3p inhibitor in LO2 cell line (*P < 0.05, Fig. 8f).

### Increased expression of circSLC3A2 was associated with poor survival in patients with HCC

**Fig. 9:**
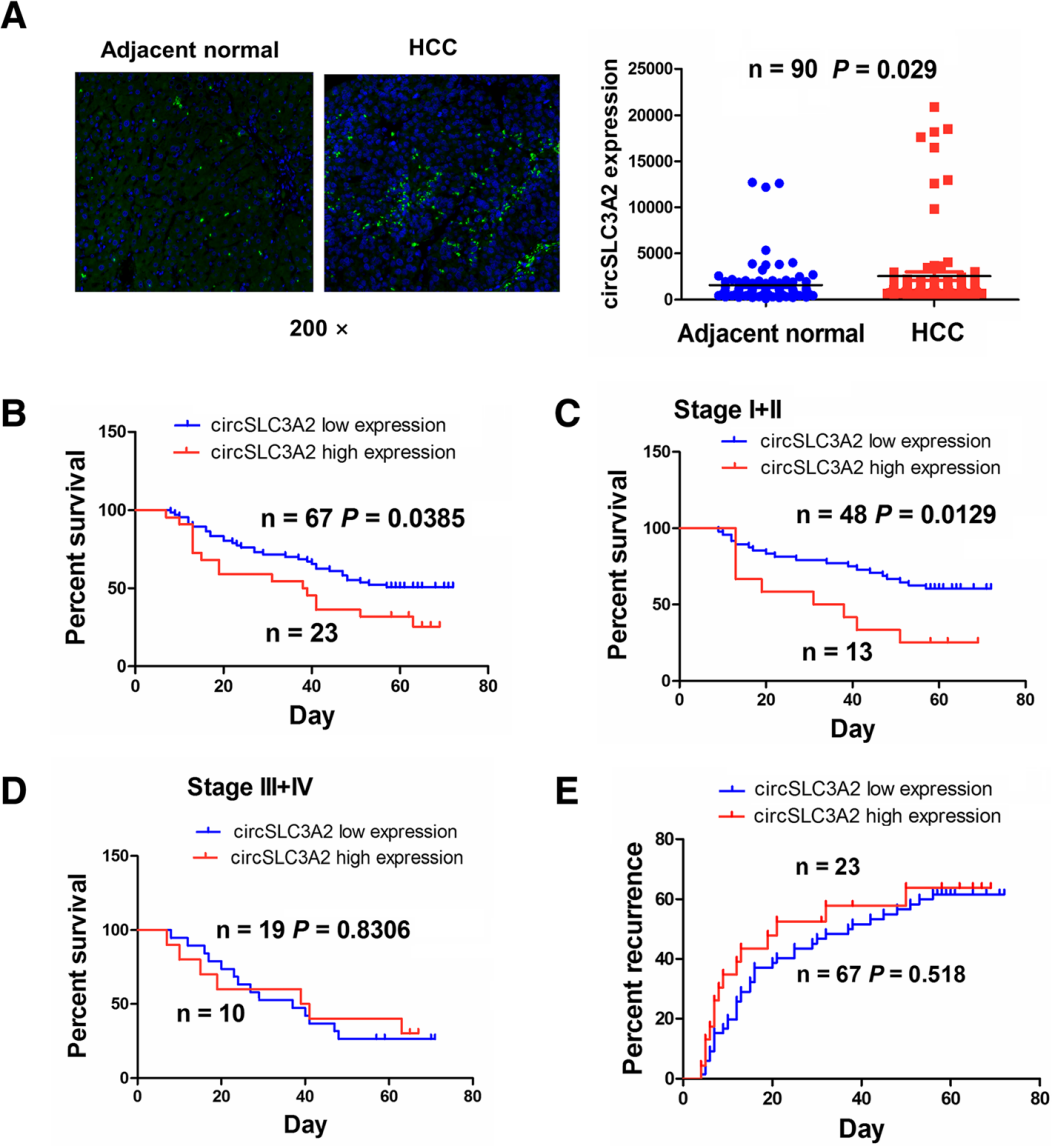
The association between circSLC3A2 expression and the prognosis in patients with HCC. **a** FISH analysis of the expression level of circSLC3A2 in HCC and adjacent normal tissues. **b** Kaplan-Meier analysis of the association of high or low circSLC3A2 expression with the overall survival in patients with HCC. **c**, **d** Kaplan-Meier analysis of the association of high or low circSLC3A2 expression with the survival in HCC patients in early and late stages. **e** Kaplan-Meier analysis of the association of high or low circSLC3A2 expression with the tumor recurrence in HCC patients

FISH analysis indicated that the expression of circSLC3A2 was elevated in HCC tissues as compared with the adjacent normal tissues (*P* = 0.020, Fig. 9a), but Pearson correlation analysis showed that circSLC3A2 had no correlation with the miR-490-3p expression in HCC tissues (*r* = − 0.1477, *P* = 0.0823, Additional file 6: Figure S5). According to the circSLC3A2 expression levels, overall survival time and status, we obtained the cutoff value of circSLC3A2 by using the cutoff finder in HCC patients, and this value divided the patients into high or low circSLC3A2 expression group. We analyzed the association of circSLC3A2 expression with clinicopathological characteristics and prognosis in patients with HCC, and found that high expression of circSLC3A2 had no association with these parameters (each *P* > 0.05) (Additional file 1: Table S11). Kaplan-Meier analysis showed that HCC patients as well as those in early stage (*P* = 0.0385, *P* = 0.0129; Fig. 9b, c) rather than in late stage (*P* = 0.8306, Fig. 9d) with high circSLC3A2 expression had the poorer survival as compared with those with low circSLC3A2 expression, but the patients with high or low circSLC3A2 expression had no difference in tumor recurrence (*P* = 0.518, Fig. 9e). **U**nivariate and multivariate Cox regression analyses revealed that circSLC3A2 expression was not an independent prognostic factors of poor survival in patients with HCC (Additional file 1: Tables S12).

## Discussion

Some studies indicate that PPM1F expression is upregulated in cancer tissues and has an association with distant metastasis and poor survival in cancer patients [5, 6]. But, our previous study showed that, PPM1F expression is downregulated in GC tissues, and loss of PPM1F expression predicts poor survival in patients with GC [8]. In this study, we found that PPM1F expression was increased in HCC tissues and high expression of PPM1F had the positive association with poor survival, acting as an independent prognostic factor in HCC patients or those in late stage. In consistence with the studies [5, 6], our results suggest that PPM1F might be a potential biomarker in HCC patients.

Furthermore, we found that, six miRNAs (miR-186-5p, miR-200c/−b, miR-429, miR-425-5p and miR-490-3p) were identified to have the potential to bind to PPM1F 3′UTR, of which miR-490 expression was dramatically decreased and had the most significant correlation with PPM1F expression in HCC tissues. Our and TCGA cohorts further confirmed that, low miR-490 expression, as an independent prognostic factor, was associated with poor survival and tumor recurrence in HCC patients and those in early or late stage. But, individual study showed that miR-490-3p is upregulated in HCC tissues and cells as compared with the adjacent non-tumor tissues [21]. In coinciding with our results, more studies revealed that miR-490-3p is downregulated in colorectal cancer (CRC) [22–24], gastric cancer (GC) [25], HCC [26] and associated with TNM stage and overall survival in CRC patients [23]. These studies indicate that miR-490-3p may be a promising diagnostic biomarker for cancer patients.

Functionally, miR-490-3p has been reported to act as a tumor suppressor in multiple malignancies. It is shown that miR-490-3p inhibits cell growth by inducing apoptosis and cell invasiveness and metastasis by repression of epithelial-to-mesenchymal transition (EMT) through targeting FRAT1, VDAC1 and TGFβR1 in CRC cells [22–24], SMARCD1 in GC [25] and BUB1 in HCC [26]. However, miR-490-3p was reported to promote cell growth and invasion via inducing EMT in HCC cells [20]. Our present studies guaranteed the consistent results with a majority of the reports [22–26] and showed the suppressive effects of miR-490-3p on HCC cell proliferation and invasion by targeting PPM1F.

**Fig. 10:**
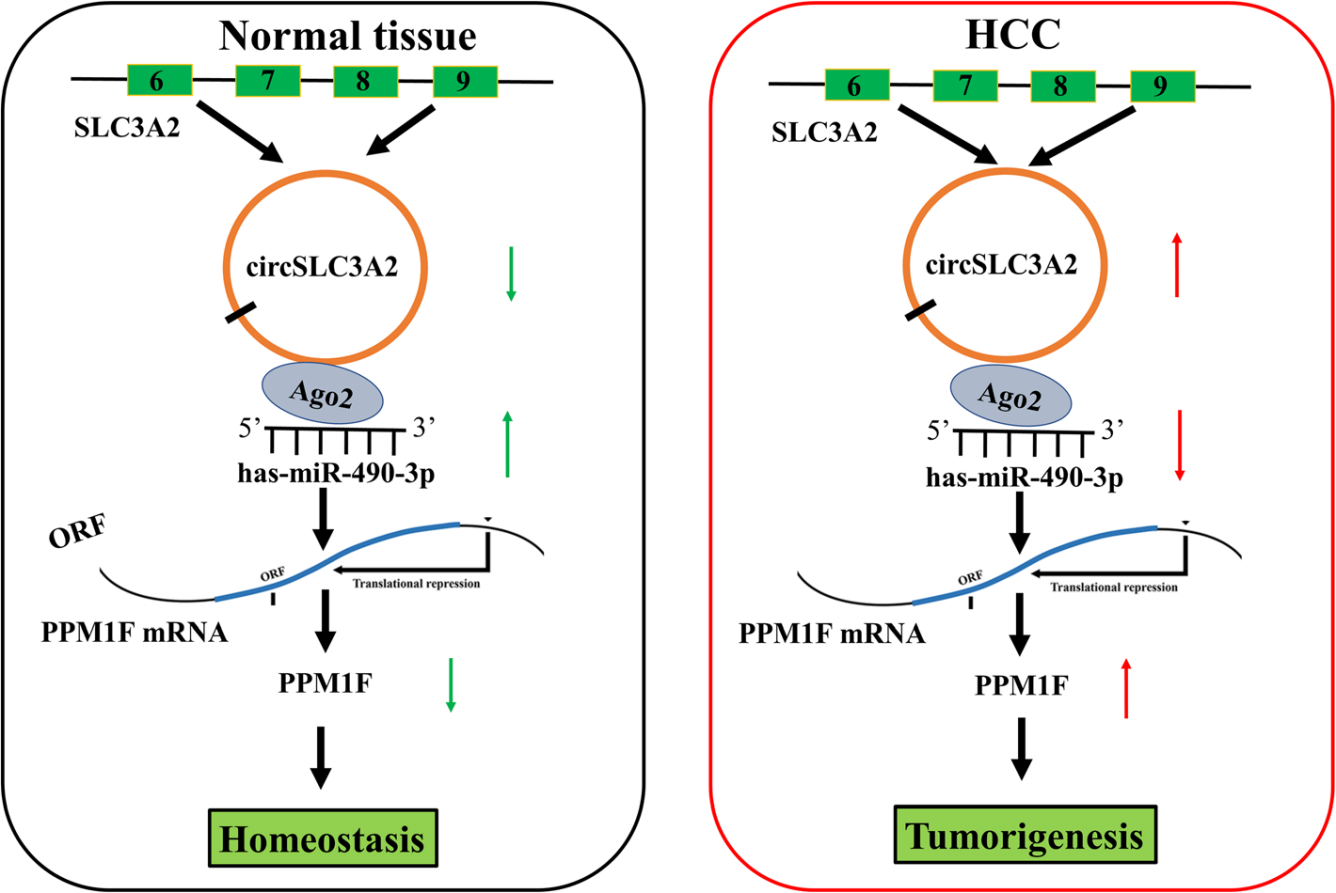
Schematic representation of the proposed mechanism of circSLC3A2 in HCC. CircSLC3A2 acted as a sponge of miR-490-3p to regulate PPM1F expression in HCC cells. Increased expression of circSLC3A2 in HCC decreased the activity of miR-490-3p, and accordingly upregulated the expression of PPM1F, leading to HCC tumorigenesis and progression

Accumulating data show that circRNAs act as miRNA sponge and induce the dysregulation of miRNAs and their target genes, leading to tumor progression including HCC [20, 27–29]. Hsa_circ_0001649 is regarded as a potential biomarker [30] and circMTO1 and circ_0067934 regulate cell growth and metastasis of HCC by sponging miR-9 and miR-1324 [18, 19]. Moreover, circ_0103809 is shown to promote HCC tumorigenesis by sponging miR-490-5p/SOX2 signaling [31]. We also identified a circRNA derived from SLC3A2 locus, named as circSLC3A2, which appeared to sponge miR-490-3p. SLC3A2 promotes integrin-dependent renal cancer growth [32] and loss of SLC3A2 inhibits Ras-driven tumorigenesis [33], Furthermore, we found that circSLC3A2, mainly localized in the cytoplasm, was upregulated in HCC tissues, associated with poor survival in patients with HCC, and facilitated cell proliferation and invasion by sponging miR-490-3p and upregulating PPM1F expression, but knockdown of circSLC3A2 reversed these effects (Fig. 10), suggesting that circSLC3A2 might function as an oncogenic factor in HCC via regulation of the miR-490-3p/PPM1F axis.

## Conclusion

Taken together, our findings demonstrated that low expression of miR-490-3p or high expression of PPM1F was positively associated with poor survival and tumor recurrence in patients with HCC, and miR-490-3p suppressed cell proliferation and invasion by targeting PPM1F. CircSLC3A2 was identified to act as an oncogenic factor in HCC by sponging miR-490-3p and regulating PPM1F expression.

## Additional files

Additional file 1: **Table S1.** List of the primer sequences. **Table S2.** The association of PPM1F expression with clinicopathologic characteristics of HCC patients. **Table S3.** Cox regression analysis of PPM1F expression as survival predictor of HCC patients. **Table S4.** Identification of miRNAs targeting PPM1F gene by microRNA.org. **Table S5.** Identification of miRNAs targeting PPM1F gene by TargetScan_7.1. **Table S6.** The association of miR-490-3p expression with clinicopathologic characteristics of HCC patients. **Table S7.** Cox regression analysis of miR-490-3p expression as survival predictor of HCC. **Table S8.** Cox regression analysis of miR-490-3p expression as recurrence predictor of HCC. **Table S9.** Identification of the circRNAs than sponge the miR-490-3p. **Table S10.** List of Ago2 occupancy in the region of circSLC3A2. **Table S11.** The association of circSLC3A2 expression with clinicopathologic characteristics of HCC patients. **Table S12.** Cox regression analysis of circSLC3A2 expression as survival predictor of HCC. (DOCX 46 kb)

Additional file 2: **Figure S1.** The association between PPM1F expression and the genetic and methylation alterations in HCC. (**A**) The genetic alterations of PPM1F in HCC. (**B**) The alterations of PPM1F in copy number in HCC. (**C**) The correlation of PPM1F expression with its DNA methylation in HCC. (PDF 1074 kb)

Additional file 3: **Figure S2.** The association between miR-490-3p expression and the prognosis in patients with HCC. (**A1–4**) Pearson’s correlation coefficient analysis of the correlation of PPM1F expression with miR-429/−200c-3p/−200b-3p/− 186-5p in HCC tissues. (**B**) ROC curve analysis of the cutoff value, sensitivity, specificity and AUC of miR-490-3p in HCC tissues in our cohort. (**C**) Kaplan-Meier analysis of the association of high or low miR-490-3p expression with the overall survival of HCC patients in late stage in our cohort. (**D**) ROC curve analysis of the cutoff value, sensitivity, specificity and AUC of miR-490-3p in HCC tissues in TCGA cohort. (PDF 2903 kb)

Additional file 4: **Figure S3.** Schematic representation of potential binding sites of miR-490-3p with the 9 circRNAs. (PDF 4573 kb)

Additional file 5: **Figure S4.** qRT-PCR analysis of the transfection efficiency of circSLC3A2 in HepG2 cells or sh-circSLC3A2 in LO2 cells. Data are the means ± SEM of three experiments. ** *P* < 0.01. (PDF 62 kb)

Additional file 6: **Figure S5.** Pearson correlation analysis of the correlation of circSLC3A2 with the miR-490-3p expression in HCC tissues. (PDF 75 kb)

## Abbreviations

AUC: Area under curve
CircRNA: Circular RNA
CRC: Colorectal cancer
FBS: Fetal bovine serum
FISH: Fluorescence in situ hybridization
GC: Gastric cancer
HCC: Hepatocellular carcinoma
IOD: Immunofluorescence accumulation optical density
MiRNA: MicroRNA
NcRNAs: Non-coding RNAs
PPM1F: Protein phosphatase Mg2+/Mn2+ dependent 1F
qRT-PCR: Quantitative real-time PCR
RIP: RNA immunoprecipitation
ROC: Receiver operating characteristic
TCGA: The Cancer Genome Atlas
TMA: Tissue microarray
