# Supplementary material for: CircSLC3A2 functions as an oncogenic factor in hepatocellular carcinoma by sponging miR-490-3p and regulating PPM1F expression
Source: Mol Cancer. 2018 Nov 23;17:165. doi: 10.1186/s12943-018-0909-7 (PMC6260990; doi:10.1186/s12943-018-0909-7)
Supplement: Supplementary file 1 — Table S1. List of the primer sequences. Table S2. The association of PPM1F expression with clinicopathologic characteristics of HCC patients. Table S3. Cox regression analysis of PPM1F expression as survival predictor of HCC patients. Table S4. Identification of miRNAs targeting PPM1F gene by microRNA.org. Table S5. Identification of miRNAs targeting PPM1F gene by TargetScan_7.1. Table S6. The association of miR-490-3p expression with clinicopathologic characteristics of HCC patients. Table S7. Cox regression analysis of miR-490-3p expression as survival predictor of HCC. Table S8. Cox regression analysis of miR-490-3p expression as recurrence predictor of HCC. Table S9. Identification of the circRNAs than sponge the miR-490-3p. Table S10. List of Ago2 occupancy in the region of circSLC3A2. Table S11. The association of circSLC3A2 expression with clinicopathologic characteristics of HCC patients. Table S12. Cox regression analysis of circSLC3A2 expression as survival predictor of HCC. (DOCX 46 kb) [file 12943_2018_909_MOESM1_ESM.docx]

**Additional file 1**

**Table S1** List of the primer sequences

| Genes | Forward | Reverse |
| --- | --- | --- |
| miR-490-3p | 5′- TGCGGTTCAAGTAATTCAGGA-3′ | 5'- CCAGTGCAGGGTCCGAGGT-3′ |
| PPM1F | 5′- GCCTACTTTGCTGTGTTTGA -3′ | 5′- TCTCGCTTGGCTTTCCT-3′ |
| CircSLC3A2 | 5′- TTTTCAGCTACGGGGATGAG -3′ | 5′- ACCTGAGTGGAGAACCACGA -3′ |
| GAPDH | 5’-AACTTTGGGATTGTGGAAGG-3’ | 5’-ACACA TTGGGGGTAGGAACA -3’ |
| U6 | 5′-GCTTCGGCAGCACATATACTAAAAT-3′ | 5′-CGCTTCACGAATTTGCGTGTCAT-3′ |

**Table S2** The correlation of PPM1F expression with clinicopathologic

characteristics of HCC patients

| Variables | Cases  (n) | PPM1F | | *P* value |
| --- | --- | --- | --- | --- |
|  |  | High | Low |  |
| Total | 340 | 114 | 226 |  |
| *Age (years)* |  |  |  |  |
| ≥60 | 174 | 64 | 110 |  |
| <60 | 166 | 50 | 116 | 0.118 |
| *Gender* |  |  |  |  |
| Male | 232 | 79 | 153 |  |
| Female | 108 | 35 | 73 | 0.432 |
| *Pathological stage* |  |  |  |  |
| Ⅰ/Ⅱ | 253 | 80 | 173 |  |
| Ⅲ/Ⅳ | 87 | 34 | 53 | 0.128 |
| *T stage* |  |  |  |  |
| T1/T2 | 256 | 81 | 175 |  |
| T3/T4 | 84 | 33 | 51 | 0.125 |
| *N stage* |  |  |  |  |
| Negative | 249 | 82 | 167 |  |
| Positive | 91 | 32 | 59 | 0.396 |
| *M stage* |  |  |  |  |
| Negative | 260 | 81 | 179 |  |
| Positive | 80 | 33 | 47 | 0.063 |

**Table S3** Cox regression analysis of PPM1F expression as survival predictor of HCC patients

| Variables | Univariate Cox regression analysis | |  | Multivariate Cox regression analysis | |
| --- | --- | --- | --- | --- | --- |
|  | RR (95% CI) | *P* value |  | RR (95% CI) | *P* value |
| *Age (years)* |  |  |  |  |  |
| <60 vs. ≥60 | 1.295 (0.894 to 1.877) | 0.171 |  | NA | NA |
| *Gender* |  |  |  |  |  |
| Male vs. Femail | 0.765 (0.525 to 1.114) | 0.162 |  | NA | NA |
| *Pathological stage* |  |  |  |  |  |
| Ⅲ/Ⅳ vs.Ⅰ/Ⅱ | 2.477 (1.707 to 3.595) | <0.0001 |  | 2.050 (0.280 to 14.990) | 0.479 |
| *T stage* |  |  |  |  |  |
| T3+T4 vs. T1+T2 | 2.500 (1.720 to 3.632) | <0.0001 |  | 1.286(0.175 to 9.430) | 0.805 |
| *N staging* |  |  |  |  |  |
| Positive vs. Negative | 1.350 (0.894 to 2.039) | 0.153 |  | NA | NA |
| *M stage* |  |  |  |  |  |
| Positive vs. Negative | 1.573 (1.045 to 2.369) | 0.030 |  | 1.619(1.070 to 2.499) | 0.023 |
| *PPM1Fexpression* |  |  |  |  |  |
| High VS. Low | 1.561(1.078 to 2.260) | 0.018 |  | 1.545(1.065 to 2.242) | 0.022 |

NA: not analyzed

|  | mRNA | mirSVR score | PhastCons score |
| --- | --- | --- | --- |
| 1 | miR-590-3p | -0.8712 | 0.776 |
| 2 | miR-186-5p | -0.9944 | 0.5882 |
| 3 | miR-200c-5p | -0.6363 | 0.5562 |
| 4 | miR-200b-5p | -0.6363 | 0.5562 |
| 5 | miR-429 | -0.6326 | 0.5562 |
| 6 | miR-506-5p | -0.6179 | 0.7116 |
| 7 | miR-124-3p | -0.6143 | 0.7116 |
| 8 | miR-425-5p | -0.5566 | 0.5882 |
| 9 | miR-499-5p | -0.4009 | 0.6556 |
| 10 | miR-490-3p | -0.4626 | 0.5882 |
| 11 | miR-135b-5p | -0.2745 | 0.7116 |
| 12 | miR-135a-5p | -0.2745 | 0.7116 |
| 13 | miR-96 | -0.2941 | 0.5882 |
| 14 | miR-1271 | -0.2915 | 0.5882 |
| 15 | miR-362-3p | -0.2618 | 0.5882 |
| 16 | miR-495 | -0.2503 | 0.5882 |
| 17 | miR-329 | -0.2488 | 0.5882 |
| 18 | miR-320d | -0.2119 | 0.6249 |
| 19 | miR-320c | -0.2119 | 0.6249 |
| 20 | miR-320b | -0.2119 | 0.6249 |
| 21 | miR-320a | -0.2119 | 0.6249 |
| 22 | miR-136 | -0.1124 | 0.7038 |
| 23 | miR-206 | -0.124 | 0.5209 |
| 24 | miR-613 | -0.124 | 0.5209 |
| 25 | miR-1 | -0.1228 | 0.5209 |
| 26 | miR-200a | -0.11 | 0.4831 |
| 27 | miR-141 | -0.1089 | 0.4831 |

**Table S4** Identification of miRNAs targeting PPM1F gene by microRNA.org

**Table S5** Identification of miRNAs targeting PPM1F gene by TargetScan_7.1

|  | miRNA | Position in the UTR | context++ score percentile | weighted context++ score |
| --- | --- | --- | --- | --- |
| 1 | hsa-miR-6778-3p | 1030-1037 | 99 | -0.31 |
| 2 | hsa-miR-2277-3p | 2078-2085 | 99 | -0.26 |
| 3 | hsa-miR-760 | 128-135 | 98 | -0.45 |
| 4 | hsa-miR-7106-5p | 28-35 | 98 | -0.3 |
| 5 | hsa-miR-6803-5p | 1092-1099 | 98 | -0.32 |
| 6 | hsa-miR-6751-5p | 1092-1099 | 98 | -0.31 |
| 7 | hsa-miR-6749-3p | 1765-1772 | 98 | -0.29 |
| 8 | hsa-miR-4308 | 696-703 | 98 | -0.37 |
| 9 | hsa-miR-429 | 866-873 | 98 | -0.21 |
| 10 | hsa-miR-425-5p | 768-775 | 98 | -0.31 |
| 11 | hsa-miR-183-5p.2 | 749-756 | 98 | -0.47 |
| 12 | hsa-miR-124-3p.1 | 941-948 | 98 | -0.3 |
| 13 | hsa-miR-362-3p | 680-686 | 98 | -0.25 |
| 14 | hsa-miR-760 | 540-547 | 97 | -0.4 |
| 15 | hsa-miR-6738-5p | 1984-1991 | 97 | -0.23 |
| 16 | hsa-miR-5194 | 1984-1991 | 97 | -0.22 |
| 17 | hsa-miR-4793-5p | 3562-3569 | 97 | -0.18 |
| 18 | hsa-miR-4744 | 807-814 | 97 | -0.26 |
| 19 | hsa-miR-4286 | 3148-3155 | 97 | -0.22 |
| 20 | hsa-miR-4283 | 1826-1833 | 97 | -0.26 |
| 21 | hsa-miR-3151-5p | 102-109 | 97 | -0.33 |
| 22 | hsa-miR-200c-3p | 866-873 | 97 | -0.2 |
| 23 | hsa-miR-200b-3p | 866-873 | 97 | -0.2 |
| 24 | hsa-miR-1914-3p | 1984-1991 | 97 | -0.19 |
| 25 | hsa-miR-329-3p | 680-686 | 97 | -0.23 |
| 26 | hsa-miR-766-3p | 2966-2973 | 96 | -0.18 |
| 27 | hsa-miR-6769b-5p | 3628-3635 | 96 | -0.19 |
| 28 | hsa-miR-6769a-5p | 3628-3635 | 96 | -0.2 |
| 29 | hsa-miR-6511b-3p | 1044-1051 | 96 | -0.25 |
| 30 | hsa-miR-6511a-3p | 1044-1051 | 96 | -0.25 |
| 31 | hsa-miR-5588-5p | 3603-3610 | 96 | -0.17 |
| 32 | hsa-miR-513b-5p | 1296-1303 | 96 | -0.16 |
| 33 | hsa-miR-4786-5p | 1317-1324 | 96 | -0.2 |
| 34 | hsa-miR-186-5p | 805-812 | 96 | -0.08 |
| 35 | hsa-miR-96-5p | 750-756 | 96 | -0.35 |
| 36 | hsa-miR-619-3p | 725-731 | 96 | -0.22 |
| 37 | hsa-miR-490-3p | 726-732 | 96 | -0.25 |
| 38 | hsa-miR-1271-5p | 750-756 | 96 | -0.32 |
| 39 | hsa-miR-6807-3p | 864-870 | 96 | -0.16 |
| 40 | hsa-miR-6791-5p | 697-703 | 96 | -0.32 |
| 41 | hsa-miR-370-3p | 1011-1017 | 96 | -0.17 |

**Table S6** The correlation of miR-490-3p expression with clinicopathologic

characteristics of HCC patients

| Variables | Cases  (n) | miR-490-3p | | *P* value |
| --- | --- | --- | --- | --- |
|  |  | High | Low |  |
| Total | 133 | 84 | 49 |  |
| *Age (years)* |  |  |  |  |
| ≥60 | 59 | 34 | 25 |  |
| <60 | 74 | 50 | 24 | 0.159 |
| *Gender* |  |  |  |  |
| Male | 97 | 61 | 36 |  |
| Female | 36 | 23 | 13 | 0.541 |
| *Pathological stage* |  |  |  |  |
| Ⅰ/Ⅱ | 101 | 63 | 38 |  |
| Ⅲ/Ⅳ | 32 | 21 | 11 | 0.455 |
| *T stage* |  |  |  |  |
| T1/T2 | 101 | 63 | 38 |  |
| T3/T4 | 32 | 21 | 11 | 0.455 |
| *N stage* |  |  |  |  |
| Negative | 101 | 66 | 35 |  |
| Positive | 32 | 18 | 14 | 0.235 |
| *M stage* |  |  |  |  |
| Negative | 100 | 63 | 37 |  |
| Positive | 33 | 21 | 12 | 0.560 |

**Table S7** Cox regression analysis of miR-490-3p expression as survival predictor of HCC

| Variables | Univariate Cox regression analysis | |  | Multivariate Cox regression analysis | |
| --- | --- | --- | --- | --- | --- |
|  | RR (95% CI) | *P* value |  | RR (95% CI) | *P* value |
| *Age (years)* |  |  |  |  |  |
| <60 vs. ≥60 | 1.975 (1.060 to 3.681) | 0.032 |  | 1.772(0.943 to 3.332) | 0.075 |
| *Gender* |  |  |  |  |  |
| Male vs. Female | 0.925 (0.487 to 1.756) | 0.811 |  | NA | NA |
| *Pathological stage* |  |  |  |  |  |
| Ⅲ/Ⅳ vs.Ⅰ/Ⅱ | 2.619 (1.408 to 4.872) | 0.002 |  | 2.788 (1.478 to 5.259) | 0.002 |
| *T stage* |  |  |  |  |  |
| T3+T4 vs. T1+T2 | 2.619 (1.408 to 4.872) | 0.002 |  | 2.788 (1.478 to 5.259) | 0.002 |
| *N staging* |  |  |  |  |  |
| Positive vs. Negative | 1.265 (0.615 to 2.604) | 0.523 |  | NA | NA |
| *M stage* |  |  |  |  |  |
| Positive vs. Negative | 2.050 (1.071 to 3.924) | 0.030 |  | 1.699(0.871 to 3.314) | 0.120 |
| *miR-490-3p expression* |  |  |  |  |  |
| High VS. Low | 0.329(1.078 to 0.608) | 0.0004 |  | 0.330(0.176 to 0.617) | 0.001 |

NA: not analyzed

**Table S8** Cox regression analysis of miR-490-3p expression as recurrence predictor of HCC

| Variables | Univariate Cox regression analysis | |  | Multivariate Cox regression analysis | |
| --- | --- | --- | --- | --- | --- |
|  | RR (95% CI) | *P* value |  | RR (95% CI) | *P* value |
| *Age (years)* |  |  |  |  |  |
| <60 vs. ≥60 | 0.816 (0.451 to 1.477) | 0.502 |  | NA | NA |
| *Gender* |  |  |  |  |  |
| Male vs. Female | 1.167 (0.601 to 2.263) | 0.649 |  | NA | NA |
| *Pathological stage* |  |  |  |  |  |
| Ⅲ/Ⅳ vs.Ⅰ/Ⅱ | 3.322 (1.785 to 6.183) | 0.0001 |  | 3.851 (2.027 to 7.317) | <0.0001 |
| *T stage* |  |  |  |  |  |
| T3+T4 vs. T1+T2 | 3.322 (1.785 to 6.183) | 0.0001 |  | 3.851 (2.027 to 7.317) | <0.0001 |
| *N staging* |  |  |  |  |  |
| Positive vs. Negative | 1.328 (0.686 to 2.571) | 0.400 |  | NA | NA |
| *M stage* |  |  |  |  |  |
| Positive vs. Negative | 1.039 (0.515 to 2.098) | 0.915 |  | NA | NA |
| *miR-490-3p expression* |  |  |  |  |  |
| High VS. Low | 0.536(0.297 to 0.966) | 0.038 |  | 0.446(0.244 to 0.816) | 0.009 |

NA: not analyzed

**Table S9** Identification of the circRNAs than sponge the miR-490-3p

| circRNA | circRNA_type | Regulation | P value | Fold change | Gene symbol | Ago2 binding sites |
| --- | --- | --- | --- | --- | --- | --- |
| hsa_circ_0022587 | exonic | UP | 0.016 | 3.587 | SLC3A2 | 2 |
| hsa_circ_0006942 | exonic | Down | 0.033 | 1.855 | ATP5H | 1 |
| hsa_circ_0053907 | exonic | Down | 0.044 | 1.618 | LTBP1 | 0 |
| hsa_circ_0004223 | exonic | Down | 0.091 | 3.494 | TCF4 | 0 |
| hsa_circ_0045006 | exonic | Down | 0.127 | 2.357 | BCAS3 | 0 |
| hsa_circ_0064557 | exonic | UP | 0.129 | 3.085 | SATB1 | 0 |
| hsa_circ_0092375 | intronic | UP | 0.079 | 2.299 | RPL27A | 12 |
| hsa_circ_0001134 | exonic | UP | 0.102 | 2.199 | XRN2 | 0 |
| hsa_circ_0009156 | exonic | UP | 0.099 | 1.909 | AP3S2 | 0 |

**Table S10** List of Ago2 occupancy in the region of circSLC3A2

| Ago2 tag name | | % Identity | | Alignment Length | | Mismatches | | Gap Openings | | Tag Start | | Tag End | circRNA Start | | circRNA ENd | |  |
| --- | --- | --- | --- | --- | --- | --- | --- | --- | --- | --- | --- | --- | --- | --- | --- | --- | --- |
| HHFCT_29233_cluster-3590_3_79_40 | | 100.00 | | 40 | | 0 | | 0 | | 1 | | 40 | | | -935 | | -896 |
| HHFKP_28061_cluster-3590_7_108_51 | | 100.00 | | 51 | | 0 | | 0 | | 1 | | 51 | | | -947 | | -897 |

**Table S11** The correlation of circSLC3A2 expression with clinicopathologic

characteristics of HCC patients

| Variables | Cases  (n) | circSLC3A2 | | *P* value |
| --- | --- | --- | --- | --- |
|  |  | High | Low |  |
| Total | 90 | 23 | 67 |  |
| *Age (years)* |  |  |  |  |
| ≥ 60 | 22 | 4 | 18 |  |
| < 60 | 68 | 19 | 49 | 0.384 |
| *Gender* |  |  |  |  |
| Male | 74 | 18 | 56 |  |
| Female | 16 | 5 | 11 | 0.567 |
| *Pathological stage* |  |  |  |  |
| Ⅰ/Ⅱ | 58 | 14 | 44 |  |
| Ⅲ/Ⅳ | 32 | 9 | 23 | 0.680 |
| *Tumor size (cm)* |  |  |  |  |
| ≥ 3 | 46 | 9 | 37 |  |
| < 3 | 44 | 14 | 30 | 0.185 |
| *TNM stage* |  |  |  |  |
| T1+T2 | 61 | 13 | 48 |  |
| T3+T4 | 29 | 10 | 19 | 0.183 |

**Table S12** Cox regression analysis of circSLC3A2 expression as survival predictor of HCC

| Variables | Univariate Cox regression analysis | |  | Multivariate Cox regression analysis | |
| --- | --- | --- | --- | --- | --- |
|  | RR (95% CI) | *P* value |  | RR (95% CI) | *P* value |
| *Age (years)* |  |  |  |  |  |
| ≥ 60 vs. < 60 | 1.164 (0.617 to 2.196) | 0.639 |  | NA | NA |
| *Gender* |  |  |  |  |  |
| Male vs. Female | 1.677 (0.753 to 3.736) | 0.206 |  | NA | NA |
| *Pathological stage* |  |  |  |  |  |
| Ⅲ/Ⅳ vs.Ⅰ/Ⅱ | 0.901 (0.501 to 1.654) | 0.757 |  | NA | NA |
| *Tumor size (cm)* |  |  |  |  |  |
| ≥ 3 vs. < 3 | 0.930 (0.530 to 1.631) | 0.800 |  | NA | NA |
| *TNM staging* |  |  |  |  |  |
| T3+T4 vs. T1+T2 | 1.934 (1.096 to 3.418) | 0.023 |  | 1.742 (0.967 to 3.135) | 0.064 |
| *CircSLC3A2 expression* |  |  |  |  |  |
| High VS. Low | 1.855 (1.018 to 3.380) | 0.043 |  | 1.608 (0.865 to 2.989) | 0.133 |

NA: not analyzed
