# Supplementary figures and images for: CircSLC3A2 functions as an oncogenic factor in hepatocellular carcinoma by sponging miR-490-3p and regulating PPM1F expression
Source: Mol Cancer. 2018 Nov 23;17:165. doi: 10.1186/s12943-018-0909-7 (PMC6260990; doi:10.1186/s12943-018-0909-7)

**A**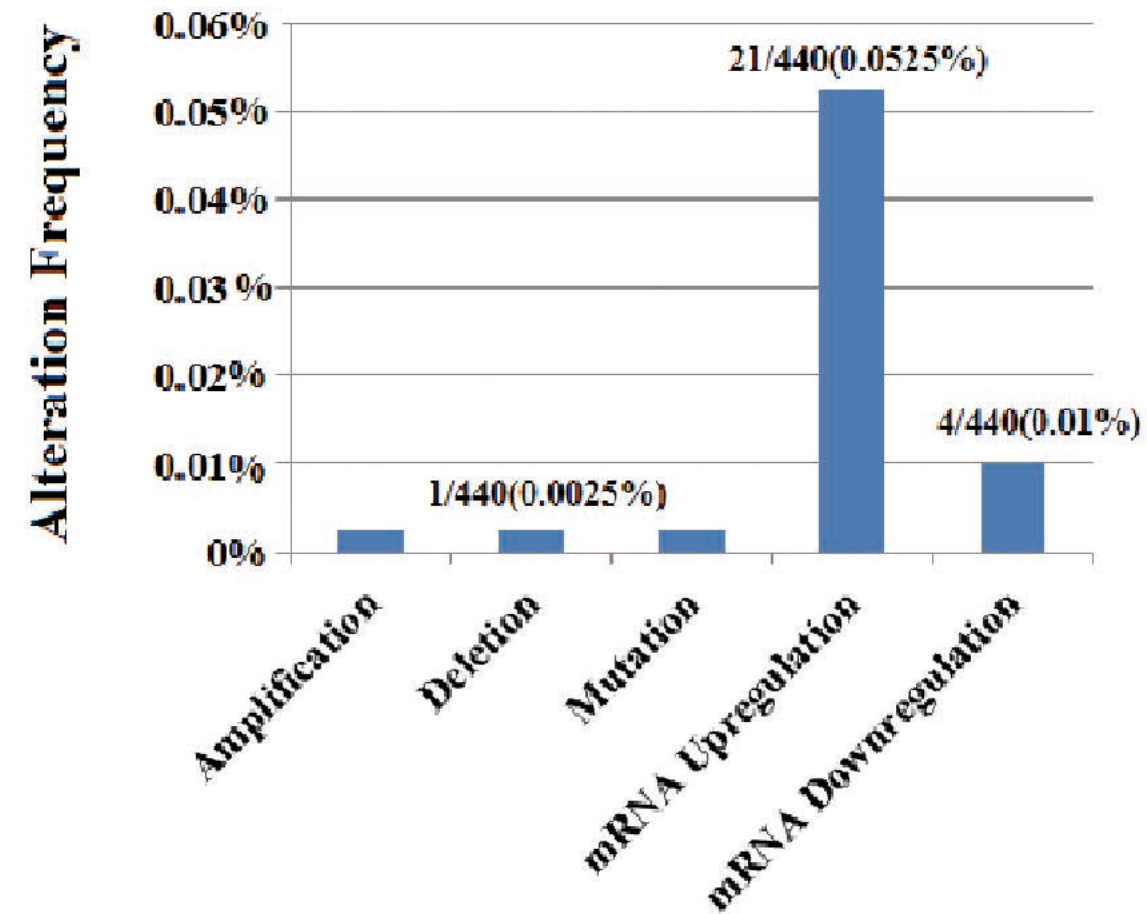**B**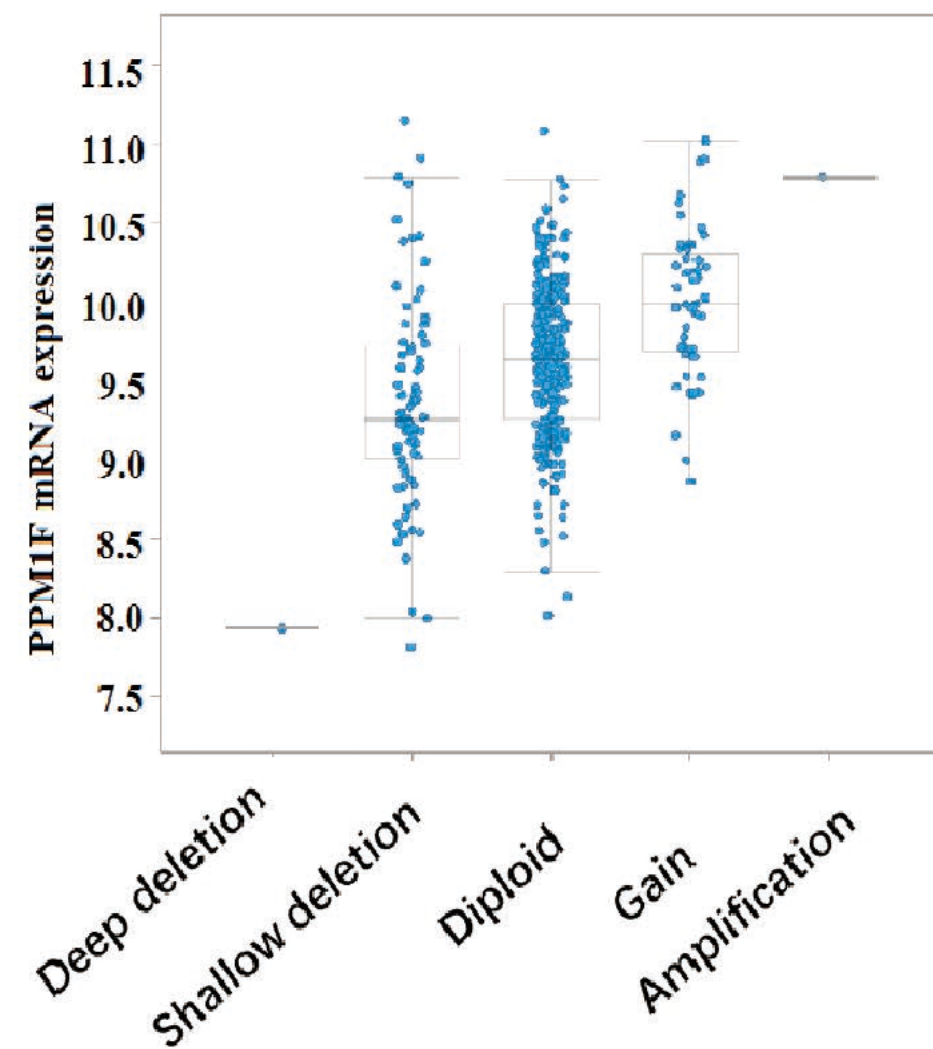**C**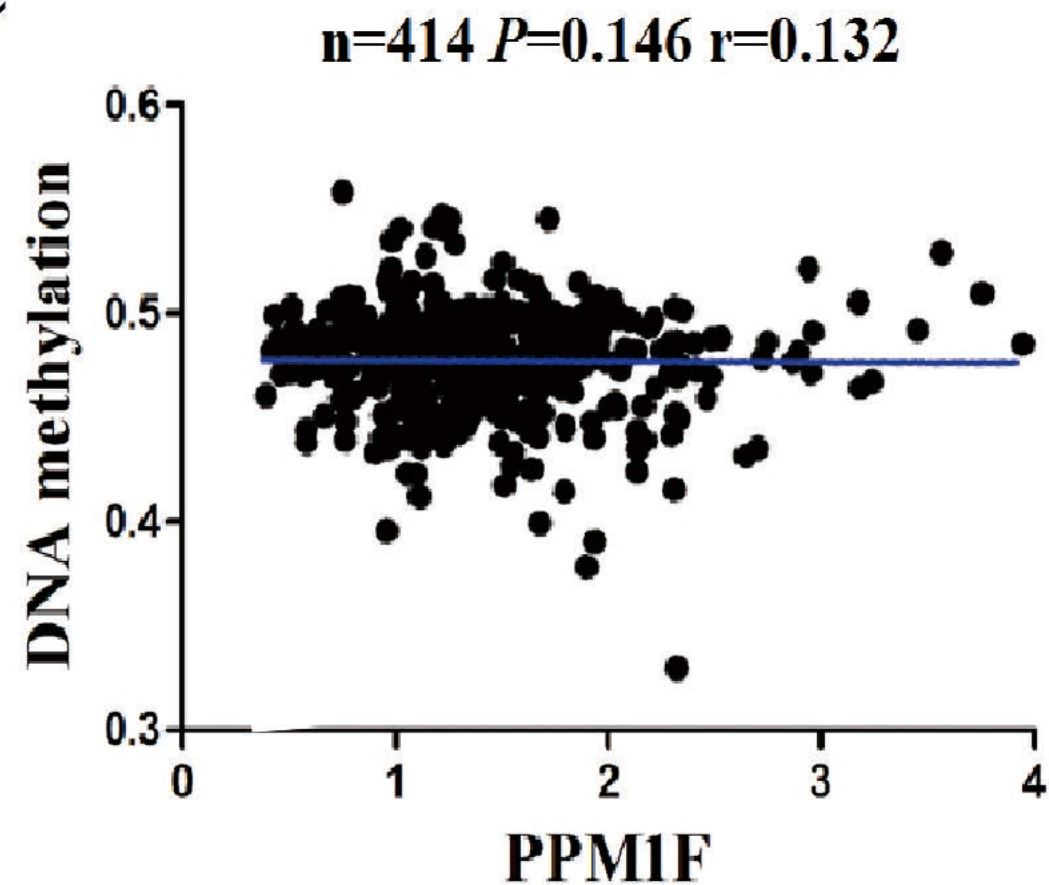

Supplement: Supplementary file 2 — Figure S1. The association between PPM1F expression and the genetic and methylation alterations in HCC. (A) The genetic alterations of PPM1F in HCC. (B) The alterations of PPM1F in copy number in HCC. (C) The correlation of PPM1F expression with its DNA methylation in HCC. (PDF 1074 kb) [file 12943_2018_909_MOESM2_ESM.pdf]

**A1**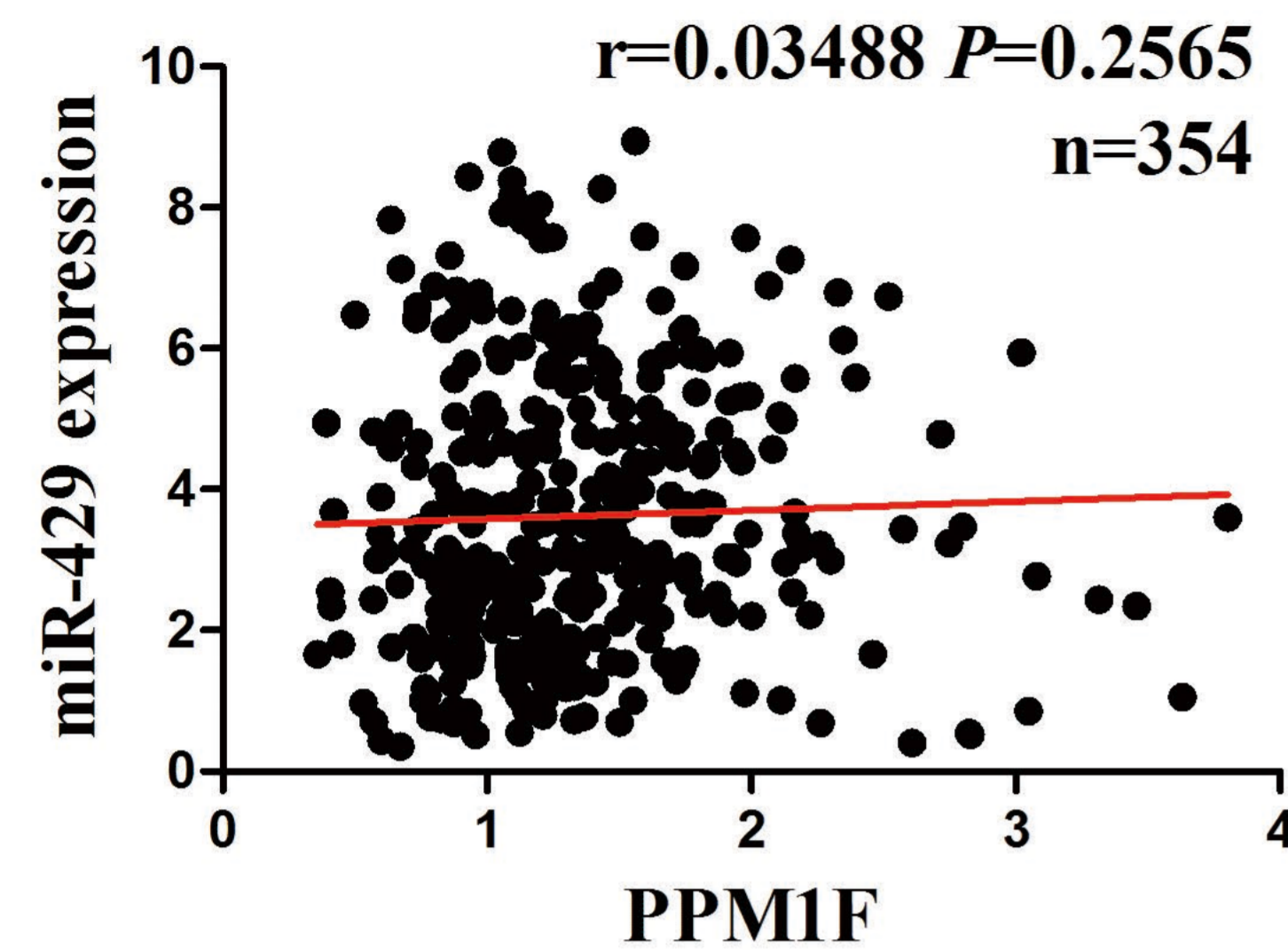**A2**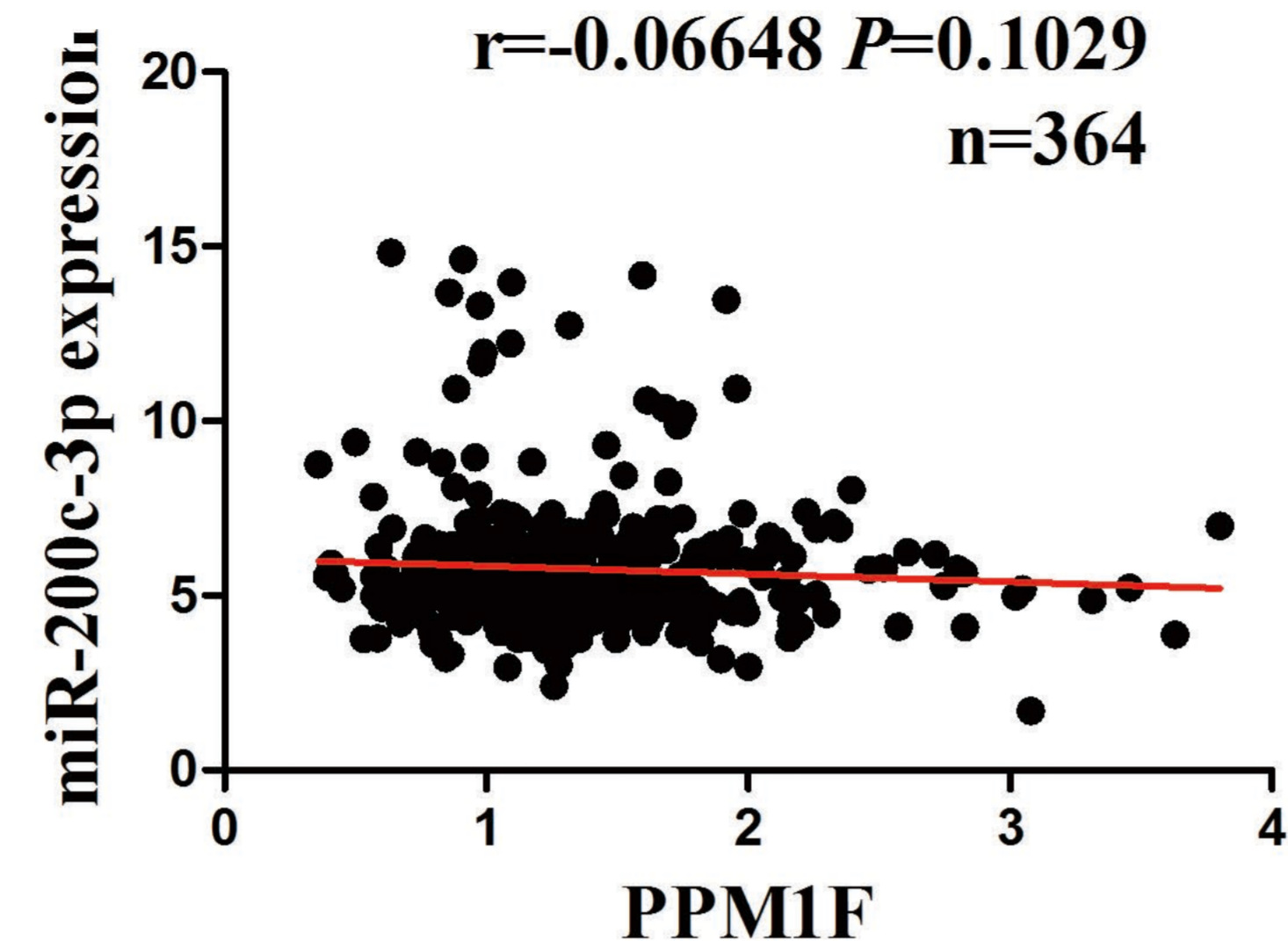**A3**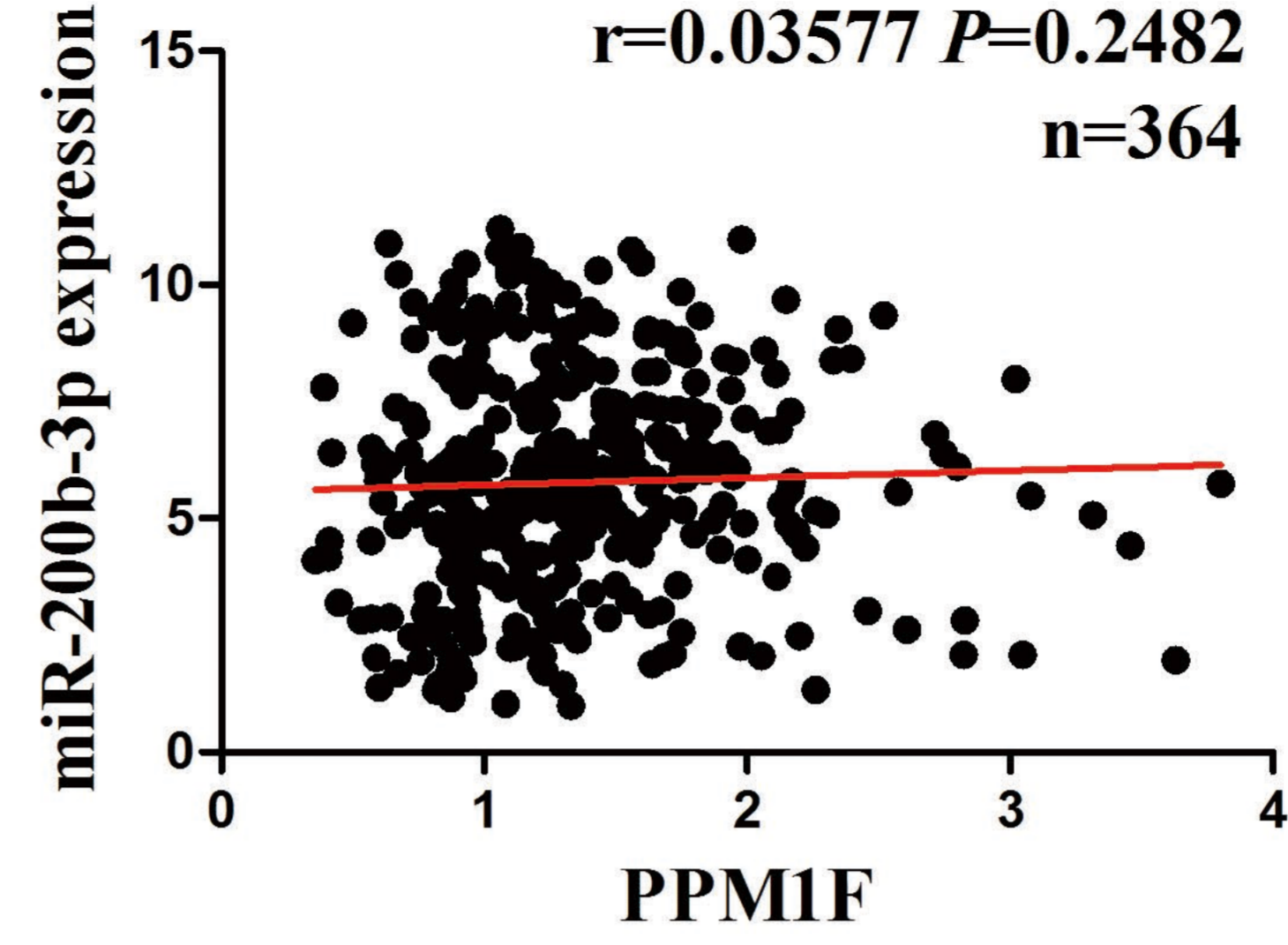**A4**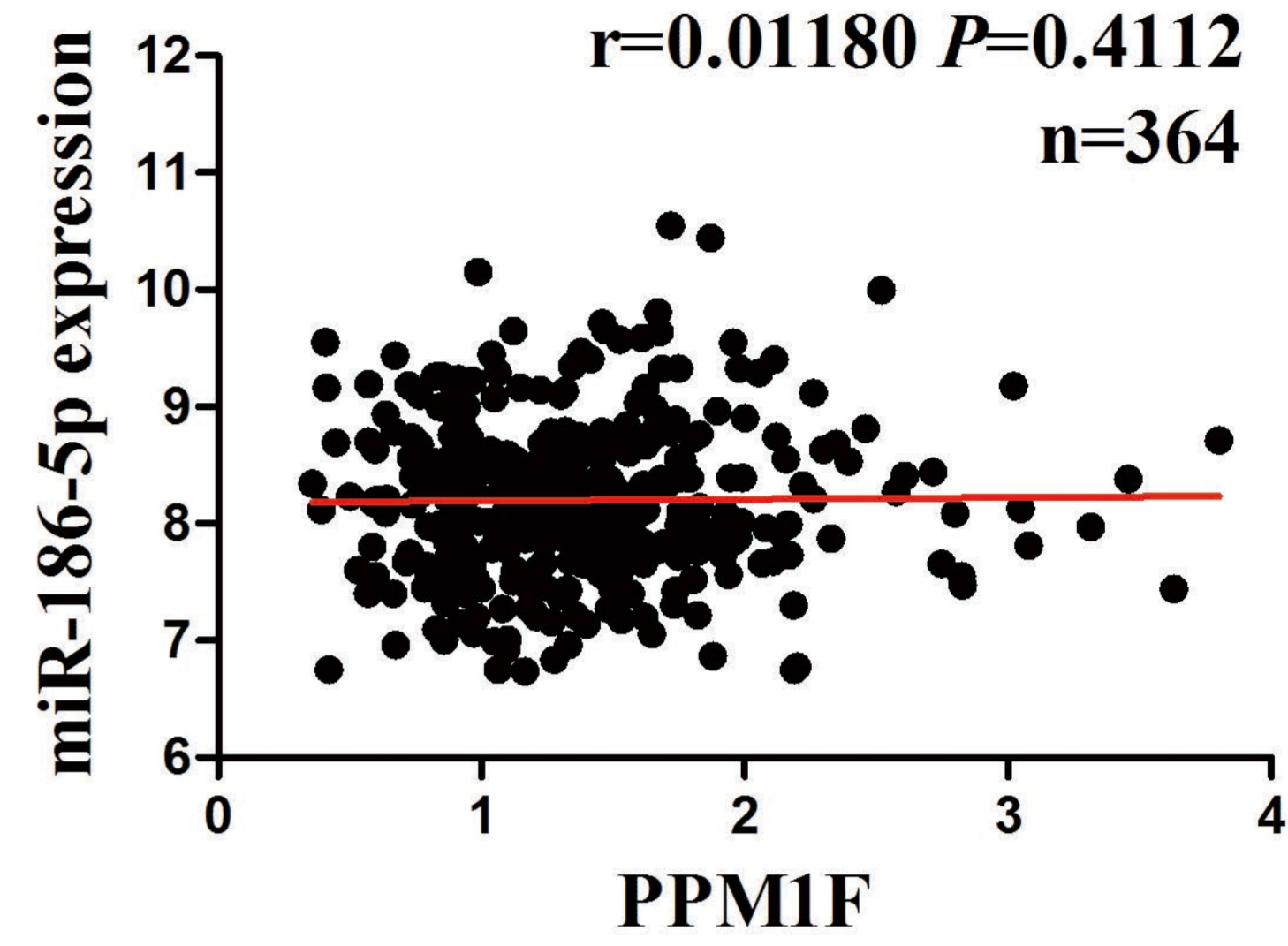**B**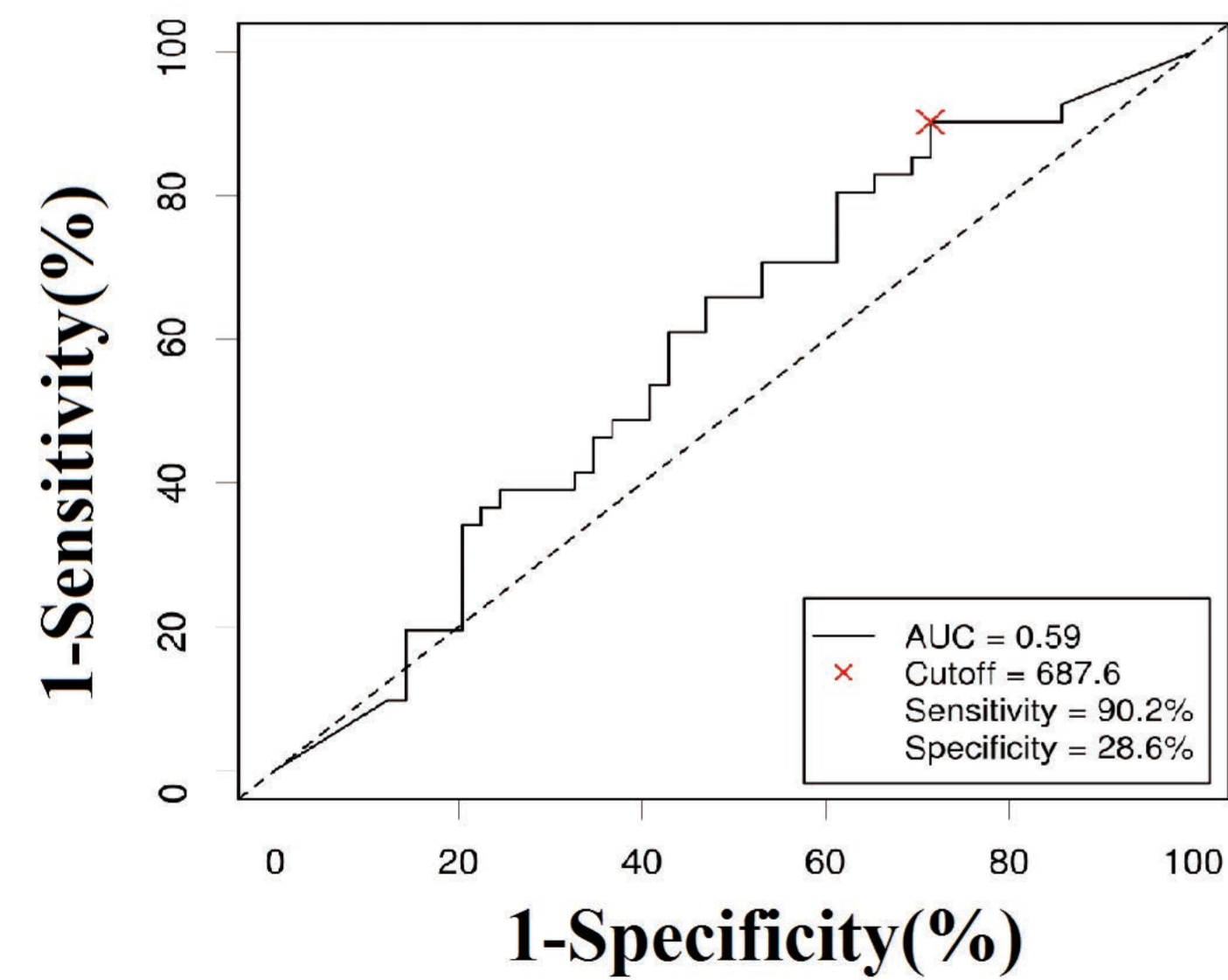**C**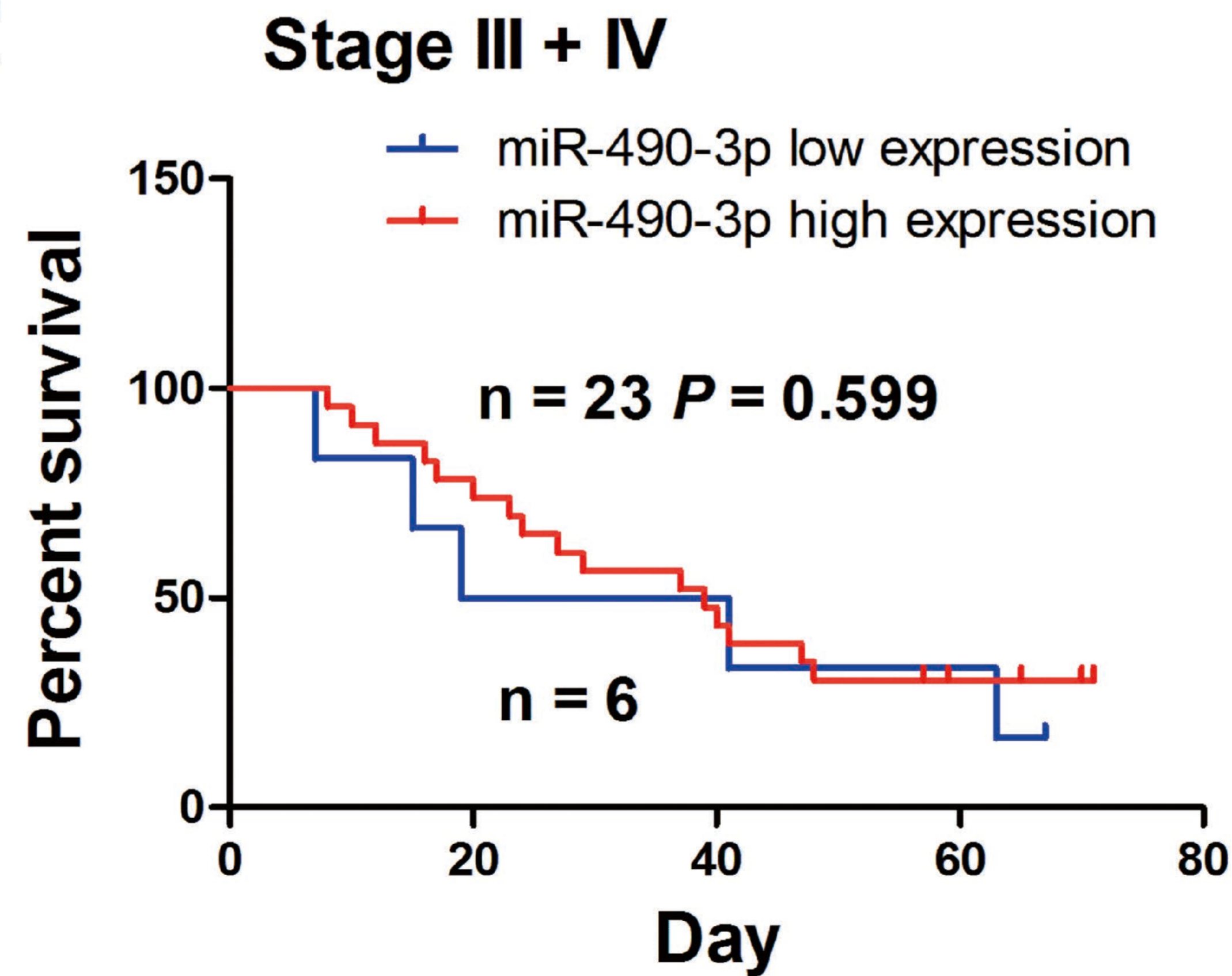**D**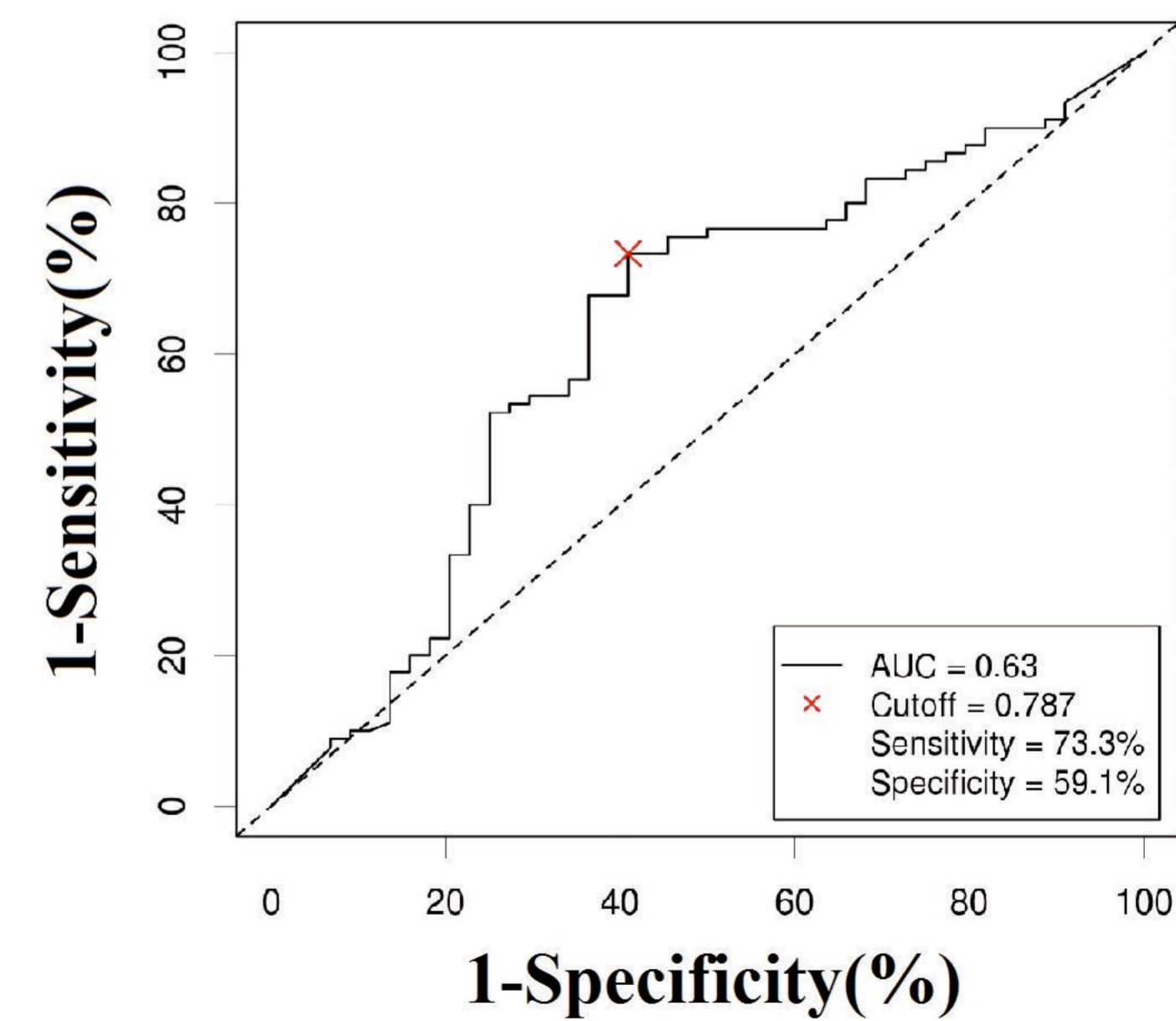

Supplement: Supplementary file 3 — Figure S2. The association between miR-490-3p expression and the prognosis in patients with HCC. (A1–4) Pearson’s correlation coefficient analysis of the correlation of PPM1F expression with miR-429/−200c-3p/−200b-3p/− 186-5p in HCC tissues. (B) ROC curve analysis of the cutoff value, sensitivity, specificity and AUC of miR-490-3p in HCC tissues in our cohort. (C) Kaplan-Meier analysis of the association of high or low miR-490-3p expression with the overall survival of HCC patients in late stage in our cohort. (D) ROC curve analysis of the cutoff value, sensitivity, specificity and AUC of miR-490-3p in HCC tissues in TCGA cohort. (PDF 2903 kb) [file 12943_2018_909_MOESM3_ESM.pdf]

circSLC3A2

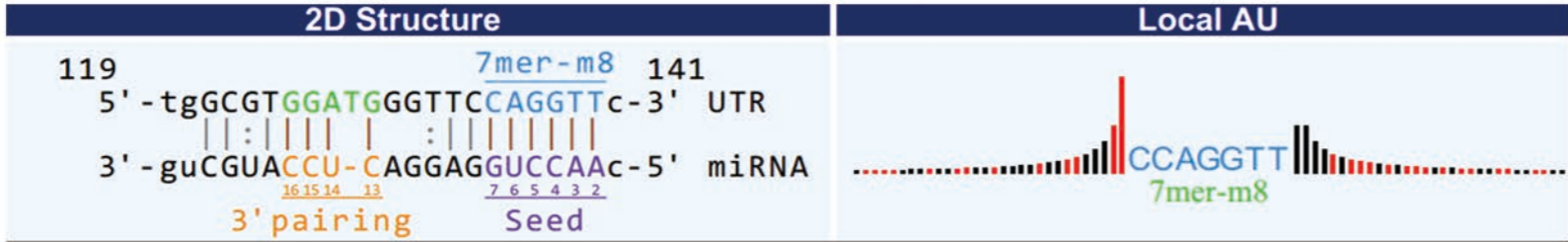

circATP5H

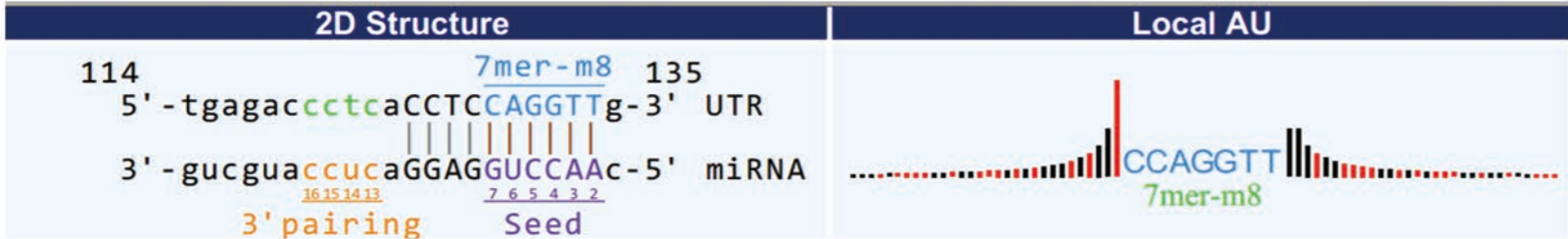

circLTBP1

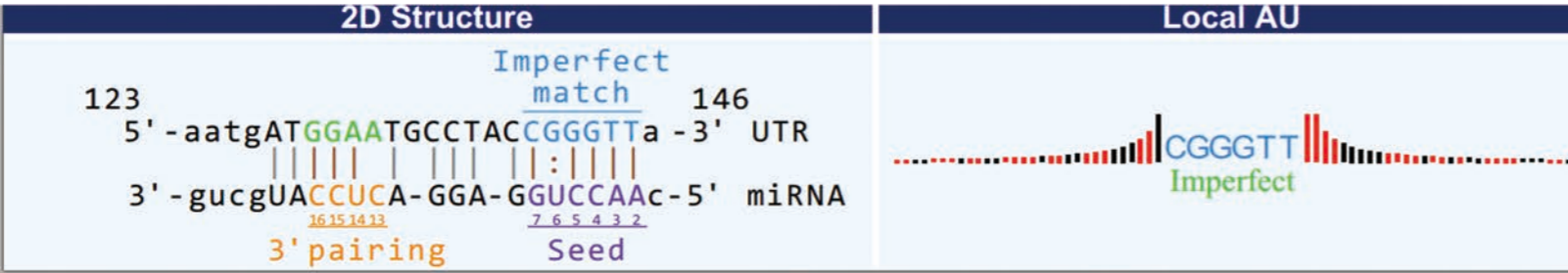

circTCF4

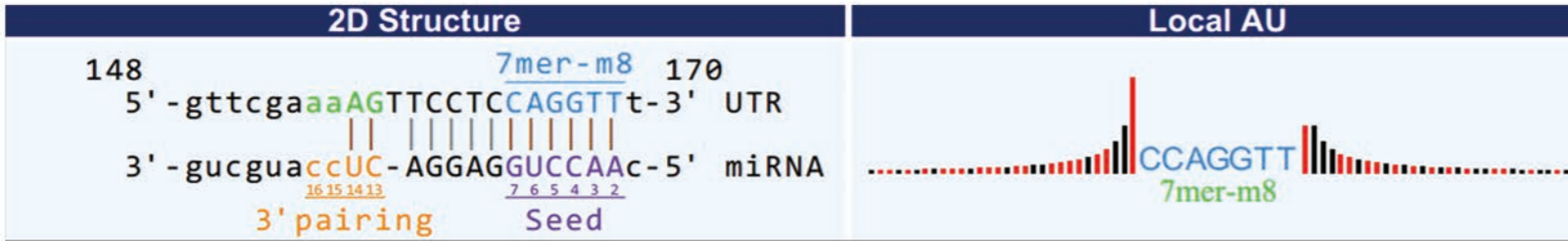

circBCAS3

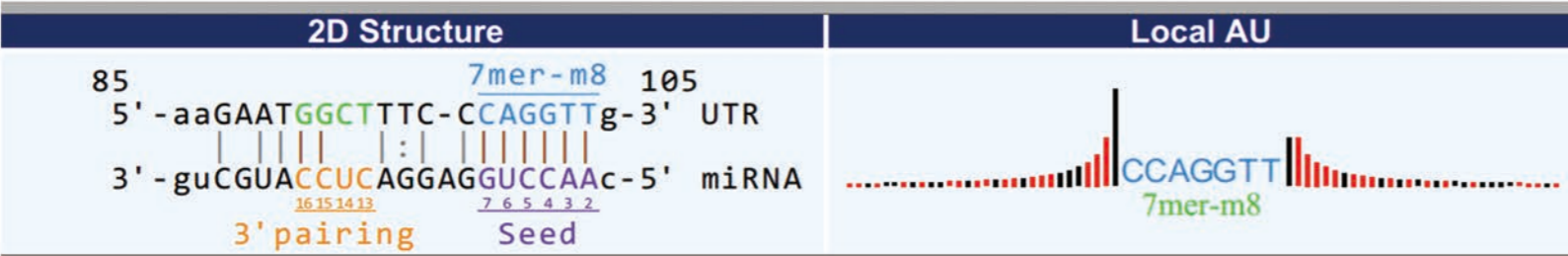

circSATB1

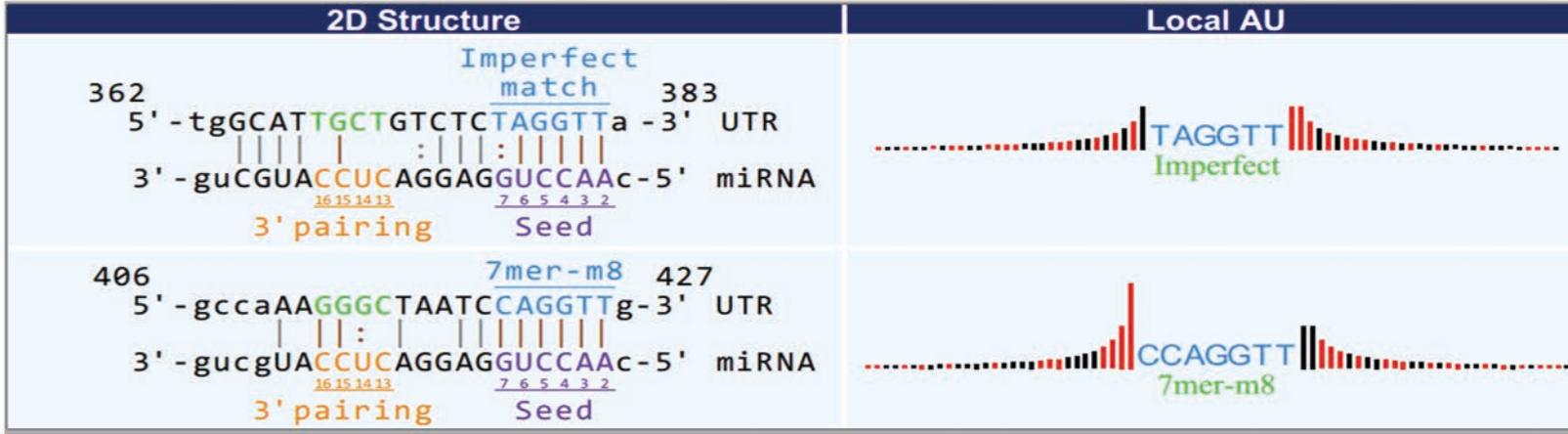

circRPL27A

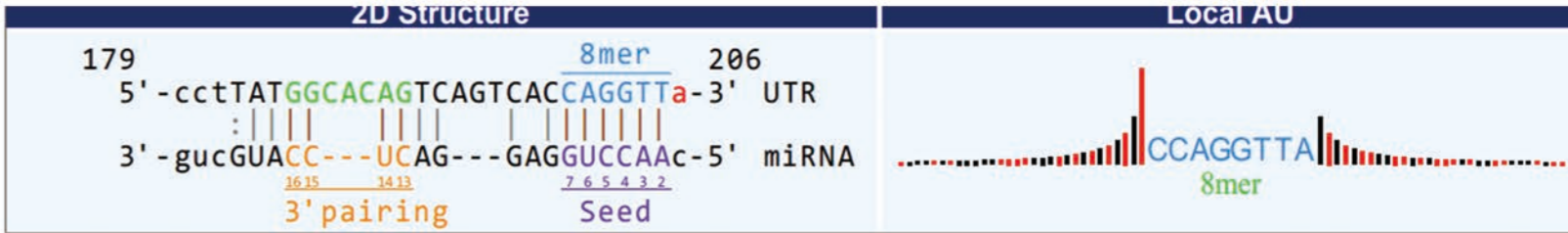

circXRN2

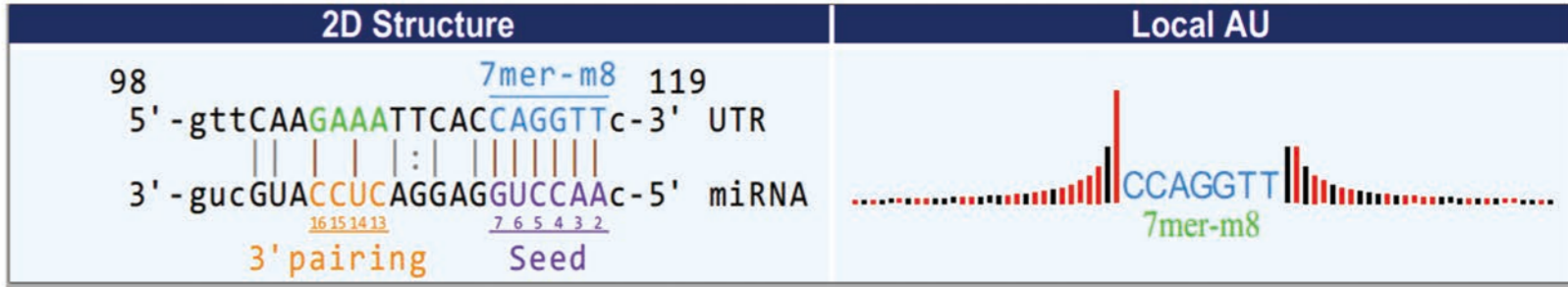

circAP3S2

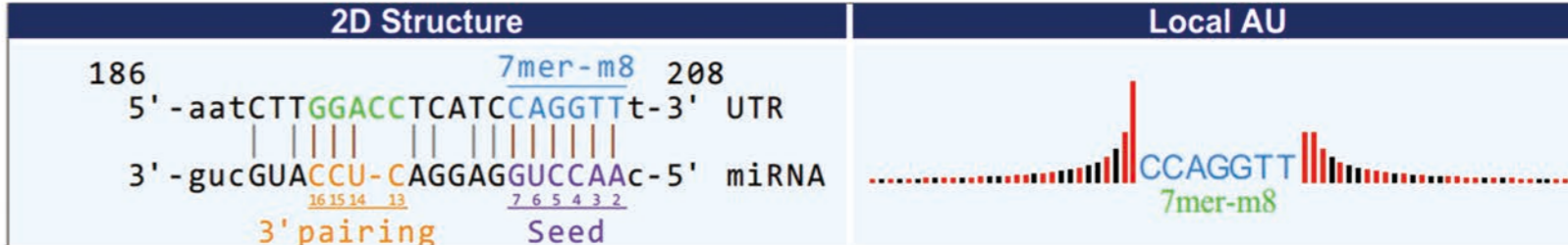

Supplement: Supplementary file 4 — Figure S3. Schematic representation of potential binding sites of miR-490-3p with the 9 circRNAs. (PDF 4573 kb) [file 12943_2018_909_MOESM4_ESM.pdf]

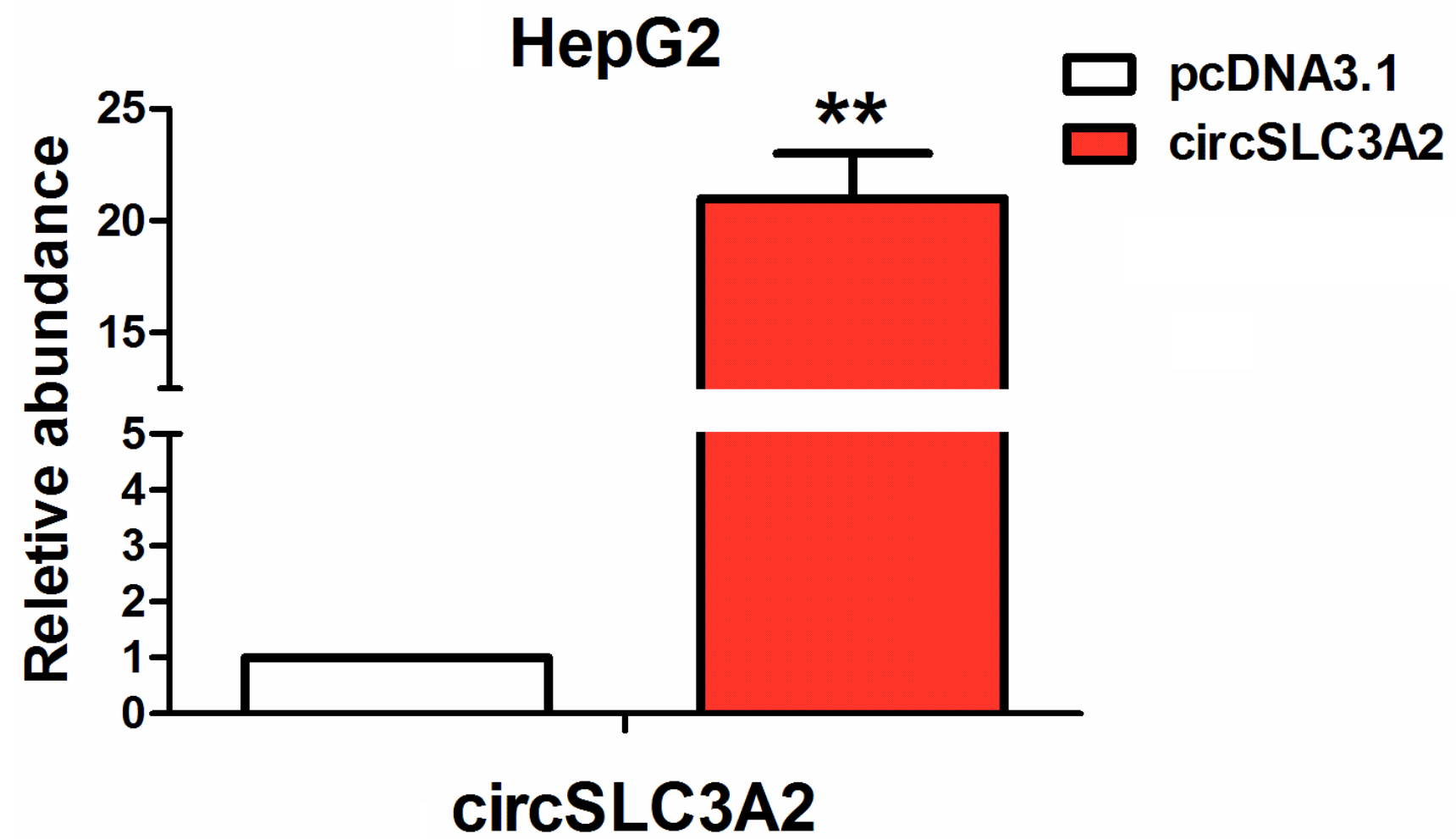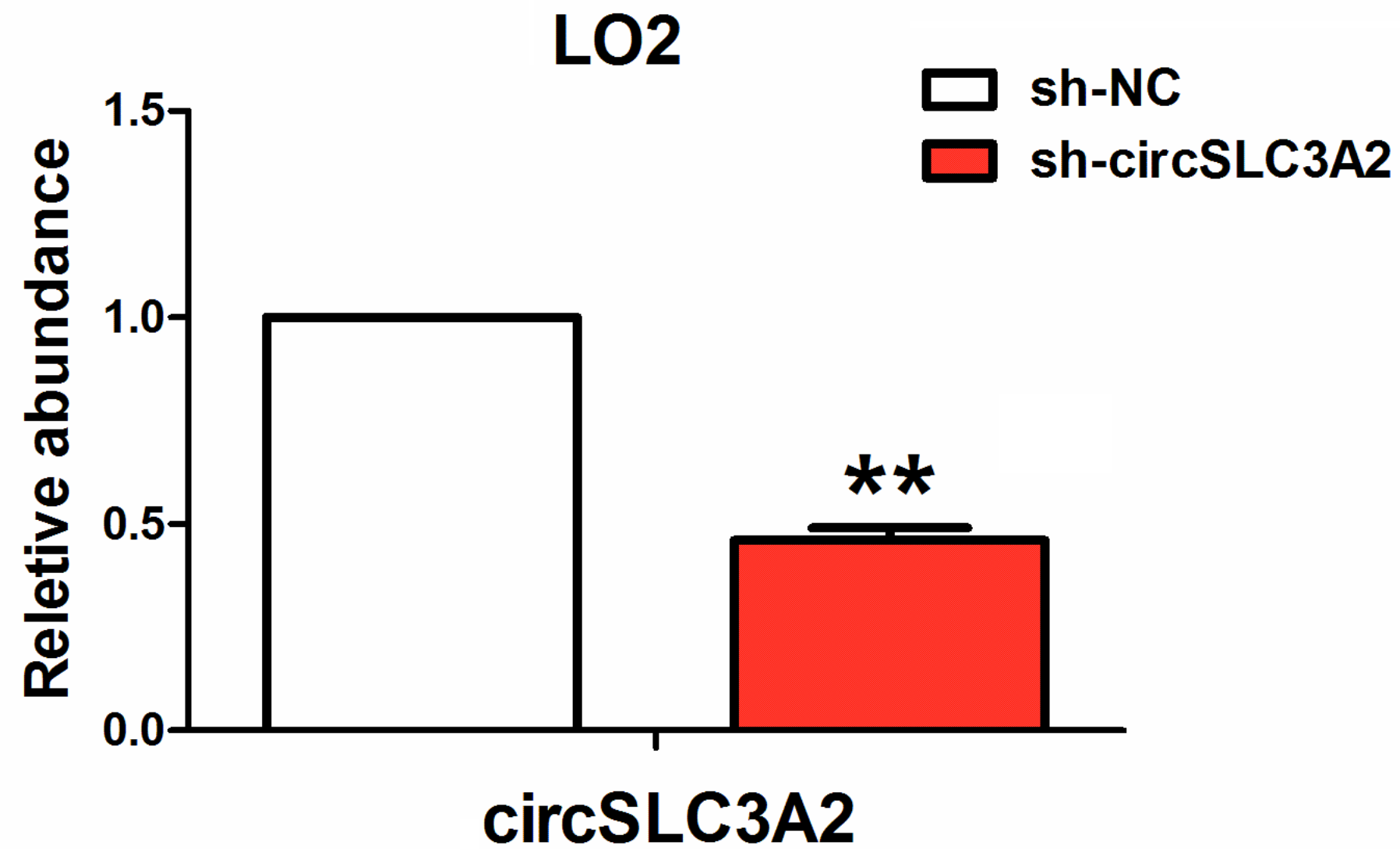

Supplement: Supplementary file 5 — Figure S4. qRT-PCR analysis of the transfection efficiency of circSLC3A2 in HepG2 cells or sh-circSLC3A2 in LO2 cells. Data are the means ± SEM of three experiments. ** P < 0.01. (PDF 62 kb) [file 12943_2018_909_MOESM5_ESM.pdf]

**CircSLC3A2 expression**

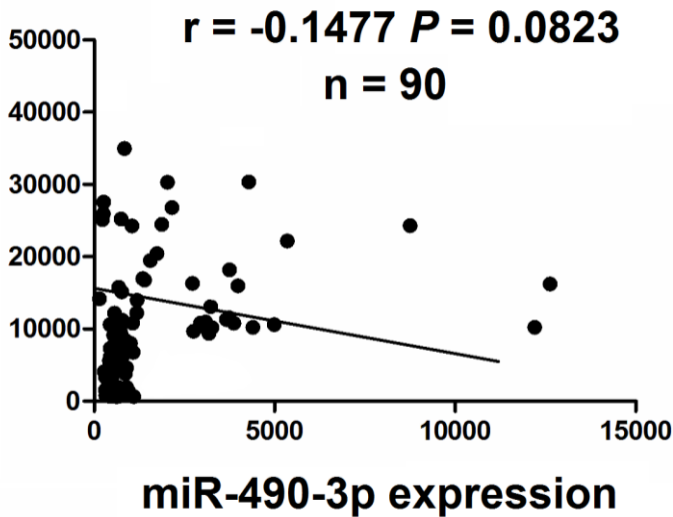

Supplement: Supplementary file 6 — Figure S5. Pearson correlation analysis of the correlation of circSLC3A2 with the miR-490-3p expression in HCC tissues. (PDF 75 kb) [file 12943_2018_909_MOESM6_ESM.pdf]
